# DROSHA is recruited to DNA damage sites by the MRN complex to promote non-homologous end joining

**DOI:** 10.1242/jcs.249706

**Published:** 2021-03-22

**Authors:** Matteo Cabrini, Marco Roncador, Alessandro Galbiati, Lina Cipolla, Antonio Maffia, Fabio Iannelli, Simone Sabbioneda, Fabrizio d’Adda di Fagagna, Sofia Francia

**Affiliations:** 1 Istituto di Genetica Molecolare, CNR – Consiglio Nazionale delle Ricerche, Pavia 27100, Italy; 2 IFOM Foundation – The FIRC Institute of Molecular Oncology Foundation, Milan 20139, Italy

**Keywords:** DNA damage, DROSHA, Non-homologous end joining, NHEJ

## Abstract

The DNA damage response (DDR) is the signaling cascade that recognizes DNA double-strand breaks (DSBs) and promotes their resolution via the DNA repair pathways of non-homologous end joining (NHEJ) or homologous recombination (HR). We and others have shown that DDR activation requires DROSHA; however, whether DROSHA exerts its functions by associating with damage sites, what controls its recruitment, and how DROSHA influences DNA repair remains poorly understood. Here, we show that DROSHA associates with DSBs independently of transcription. Neither H2AX, nor ATM or DNA-PK kinase activities are required for recruitment of DROSHA to break sites. Rather, DROSHA interacts with RAD50, and inhibition of the MRN complex by mirin treatment abolishes this interaction. MRN complex inactivation by RAD50 knockdown or mirin treatment prevents DROSHA recruitment to DSBs and, as a consequence, also prevents 53BP1 (also known as TP53BP1) recruitment. During DNA repair, DROSHA inactivation reduces NHEJ and boosts HR frequency. Indeed, DROSHA knockdown also increases the association of downstream HR factors such as RAD51 to DNA ends. Overall, our results demonstrate that DROSHA is recruited at DSBs by the MRN complex and directs DNA repair towards NHEJ.

## Abstract (precis)

**Summary:** DROSHA is part of the primary response to DNA damage and is recruited at double-strand breaks in an MRN-dependent manner. DROSHA fosters NHEJ repair and inhibits HR by controlling 53BP1 recruitment.

## INTRODUCTION

DNA lesions constantly challenge genome integrity, and efficient DNA repair is crucial to avoid genome instability leading to pathological processes such as cancer and aging. The DNA damage response (DDR) is the cascade of events that detects, signals and coordinates repair of DNA lesions. DNA double-strand breaks (DSBs) represent one of the major activators of this pathway. These lesions are recognized by the MRE11–RAD50–NBS1 (NBN) sensor complex (MRN complex), which recruits and activates ataxia telangiectasia mutated protein kinase (ATM; Shiloh, 2006). The MRN complex exerts different functions at DNA damage sites, including DNA clipping, 3′-5′ DNA exonuclease (Bian et al., 2019; Nicolette et al., 2010) and DNA melting activities by transiently separating the strands of the DNA duplex (Cannon et al., 2013). These events trigger activation of ATM by phosphorylation (pATM) as well as the local phosphorylation of histone H2AX (γH2AX). Upon γH2AX spreading along the chromosome, DDR mediators, such as MDC1 and 53BP1 (also known as TP53BP1), are recruited leading to the formation of cytologically detectable DDR foci (Ciccia and Elledge, 2010). The accumulation of DDR factors to DSBs has been described as a two-step process. The initial recruitment occurs by a direct recognition of the DNA lesion in a γH2AX-independent manner (primary recruitment), followed by retention of DDR proteins at the damaged site in a γH2AX-dependent manner (secondary recruitment) (Celeste et al., 2003). DSBs also directly recruit the KU70–KU80 (XRCC6–XRCC5) heterodimer, which instead activates the DNA-dependent protein kinase catalytic subunit (DNA-PKcs, also known as PRKDC) involved in DNA repair by non-homologous end joining (NHEJ) in a process in which DNA ends are directly ligated (Blackford and Jackson, 2017). Poly(ADP-ribose) polymerase 1 (PARP1) is also rapidly activated by DSBs, where it poly(ADP-ribosyl)ates itself and other chromatin-associated proteins to signal the presence of the lesion and to stimulate DNA repair (Ray Chaudhuri and Nussenzweig, 2017).

We previously reported the generation of a novel class of DROSHA- and DICER-dependent small non-coding RNA, named DNA damage response RNAs, or DDRNAs, involved in local DDR activation by promoting the secondary recruitment of DDR mediators 53BP1 and MDC1 (d'Adda di Fagagna, 2014; Francia, 2015; Francia et al., 2016, 2012). Our results suggest a model in which DSBs are first recognized by the DNA damage sensor MRN, which then recruits RNA polymerase II (RNAP II) together with the pre-initiation complex (PIC) (Pessina et al., 2019). Transcription at the damaged site leads to the synthesis of damage-induced long ncRNAs (dilncRNAs), which upon processing by DROSHA and DICER generate DDRNAs (Michelini et al., 2017). Recently, we showed that an *in vitro* reconstitution of the minimal apparatus for dilncRNA transcription, made of MRN and RNAP II, allows us to recapitulate the transcriptional events occurring at DSBs (Sharma et al., 2021). Both dilncRNAs and DDRNAs interact with DDR factors such as 53BP1 and nucleate the focal accumulation of 53BP1 via their phase separation into liquid-like droplets (Pessina et al., 2019).

DICER, in its phosphorylated form, has been recently reported to associate with DSBs and mediate 53BP1 foci formation (Burger and Gullerova, 2018; Burger et al., 2017), and in plants, DICER-dependent ncRNAs, similar in structure to DDRNAs, play a role in DNA repair by homologous recombination (HR) (Wei et al., 2012) and NHEJ (Gao et al., 2014). DROSHA has been proposed to be recruited at DNA breaks to promote the formation of DNA–RNA hybrids required for HR (Lu et al., 2018).

Here we show, by a genome-wide approach, that DROSHA associates with DSBs occurring at endogenous sequences irrespective of their transcriptional status. DROSHA recruitment is independent of the presence of γH2AX and of ATM and DNA-PKcs kinase activities, occurring as one of the most upstream events in the DDR cascade. We also show that DROSHA interacts with RAD50 and that this interaction is reduced upon inhibition of MRN activity by the small-molecule inhibitor mirin. Importantly, MRN chemical inhibition and RAD50 knockdown reduces DROSHA association with DSBs, suggesting that DROSHA interaction with the MRN complex promotes recruitment. Finally, we show that DROSHA plays an important role in DNA repair by NHEJ, whereas it is dispensable for HR. Indeed, lack of DROSHA results in a reduced level of 53BP1 at break sites, thus releasing DNA ends for more RAD51 recruitment at HR-prone sites.

## RESULTS

### DROSHA is recruited to DSBs

DROSHA is known to localize predominantly in the nucleus and to work co-transcriptionally in the context of microRNA processing (Gromak et al., 2013; Morlando et al., 2012). Given the role of DROSHA in processing newly synthetized dilncRNAs into DDRNAs, we investigated whether DROSHA is recruited to sites of DNA damage. To test this hypothesis, we took advantage of the DIvA (DSB inducible via AsiSI) cellular system (Aymard et al., 2017, 2014; Capozzo et al., 2017; Iacovoni et al., 2010), a clonal U2OS cell line that stably expresses the AsiSI restriction enzyme fused to a modified oestrogen receptor ligand-binding domain. Treatment of cells with 4-hydroxytamoxifen (4OHT) triggers nuclear localization of the AsiSI enzyme and the generation of several chromosomal breaks per cell that have been accurately mapped (Aymard et al., 2017, 2014; Capozzo et al., 2017; Caron et al., 2015; Iannelli et al., 2017) (Fig. S1A). This cellular system is ideal for use in chromatin immunoprecipitation (ChIP) assays, which can be analysed by either next-generation sequencing (ChIP-seq) or real-time quantitative PCR (ChIP-qPCR), to identify the association of DROSHA with sites of DNA damage across the genome. We previously identified 50 AsiSI sites robustly and reproducibly cut among independent experiments (top 50 AsiSI sites) by performing ChIP-seq analyses for γH2AX and combining the resulting list of AsiSI sites with one obtained using a breaks labeling *in situ* and sequencing (BLISS) approach (Iannelli et al., 2017; Yan et al., 2017).

**Fig. 1.**
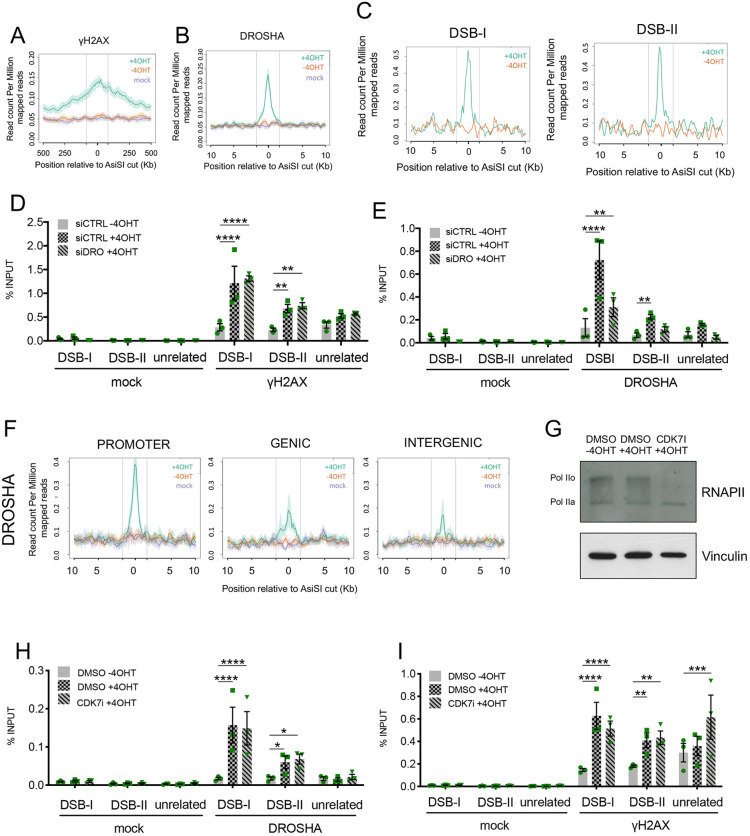
**DROSHA accumulates at sites of DNA damage.** (A) Mean±s.e.m. γH2AX ChIP-seq signals of the 50 most cut AsiSI sites, over 1 Mb windows and centered at the AsiSI site, are shown for cut (+4OHT, green), uncut (−4OHT, red) or mock (magenta) samples. (B) Mean±s.e.m. DROSHA ChIP-seq signals of the 50 most cut AsiSI sites, over 20 kb windows and centered at the AsiSI site, are shown for cut (+4OHT, green), uncut (−4OHT, red) or mock (magenta) samples. (C) Coverage plot profile representing the read count per million mapped reads (RPM) of DROSHA ChIP-seq for cut (+4OHT, green) and uncut (−4OHT, red) samples at two representative AsiSI sites, DSB-I and DSB-II, among the most cut (Iannelli et al., 2017). Vertical lines in A–C indicate the boundaries of the region used to center all the top 50 AsiSI sites. (D,E) The bar plots show the percentage of ChIP enrichment, relative to the input, of mock (no antibody) and of γH2AX (D) or DROSHA (E) as detected by ChIP-qPCR in cut (+4OHT) and uncut (−4OHT) DIvA cells treated with control siRNA (siCTRL), and in cut DIvA cells knocked down for DROSHA (siDRO +4OHT), with primers targeting DSB-I, DSB-II or an unrelated genomic region far from any annotated AsiSI sites. Data are mean±s.e.m. from three independent experiments. **P≤0.01; ****P≤0.0001 (two-way ANOVA with Tukey's multiple comparison test). (F) Mean±s.e.m. DROSHA ChIP-seq signals of eight AsiSI sites positioned in regions annotated either as a promoter, genic or intergenic region, over 20 kb windows and centered at the AsiSI site, are shown for cut (+4OHT, green), uncut (−4OHT, red) or mock (magenta) samples. All the AsiSI sites assayed are included in a previously published set of most cut AsiSI sites (Iannelli et al., 2017). Vertical lines indicate the boundaries of the region used to center all the top 50 AsiSI sites. (G) DIvA cells were treated with CDK7 inhibitor (CDK7I) or mock treated with DMSO. Phosphorylation status of RNAP II was evaluated by western blotting. Pol IIo indicates hyperphosphorylated CTD, Pol IIa indicates unphosphorylated CTD. Vinculin was used as loading control. (H,I) The bar plots show the percentage of ChIP enrichment, relative to the input, of mock (no antibody) and of DROSHA (H) or γH2AX (I) as detected by ChIP-qPCR in cut (+4OHT) and uncut (−4OHT) DIvA cells mock treated with DMSO, and in cut DIvA cells treated with CDK7 inhibitor (CDK7i), with primers matching DSB-I, DSB-II or an unrelated genomic region far from any annotated AsiSI sites Data are mean±s.e.m. from three independent experiments. *P≤0.05; **P≤0.01; ***P≤0.001; ****P≤0.0001 (two-way ANOVA with Tukey's multiple comparison test).

As previously reported in this cellular system (Aymard et al., 2014; Iacovoni et al., 2010; Iannelli et al., 2017), the averaged γH2AX ChIP-seq signal of the top50 AsiSI sites showed a megabase-wide region of modified chromatin spreading away from the DSBs (Fig. 1A; Fig. S1B).

Strikingly, the DROSHA ChIP-seq averaged signal at the same DSBs resulted in a sharp and narrow peak focused around the AsiSI cut sites, indicating a clear and localized recruitment of the protein upon damage generation (Fig. 1B; Fig. S1C). DROSHA accumulation upon DSB induction was clearly detected also when analyzed at individual AsiSI sites (DSB-I and DSB-II) selected among the top 50 (Fig. 1C). It is worth observing that the ChIP-seq profile of DROSHA is similar to that observed for the NHEJ factor X-ray repair cross-complementing protein 4 (XRCC4) in the same cellular system (Aymard et al., 2014).

To validate these observations and to confirm the specificity of the signal observed, we performed ChIP-qPCR analysis at DSB-I and DSB-II. As expected, both γH2AX and DROSHA signals increased upon 4OHT administration (Fig. 1D,E), and DROSHA signal was substantially reduced in cells knocked down for DROSHA (Fig. 1E; Fig. S1D), despite exhibiting robust γH2AX (Fig. 1D), as previously observed (Francia et al., 2016, 2012; Michelini et al., 2017). Moreover, ChIP-qPCR with primers mapping at increasing distances from the DSB-II site (60 bp, 1 kb and 2.5 kb) confirmed that DROSHA enrichment, differently from γH2AX, peaks near the DSB and does not spread on the surrounding chromatin (Fig. S1E,F).

To investigate whether the transcriptional status in which the DSB occurs affects DROSHA recruitment, we identified – among the top 50 AsiSI sites – three groups of DSBs lying in different genetic contexts: (1) promoters, (2) genic regions or (3) intergenic regions. Due to the prevalence in CG content of the AsiSI consensus site (5′-GCGATCGC-3′), promoters are privileged areas for their presence (Iannelli et al., 2017). Indeed, 26 of the 50 sites mapped to a promoter region, whereas only eight sites were classified as intergenic (Fig. S1G). Thus, to operate a fair comparison we considered only eight AsiSI sites for each group. All of them showed a robust γH2AX signal, indicating a comparable cut efficiency (Fig. S1H). Interestingly, DROSHA recruitment to AsiSI DSBs occurred independently of genetic context, although the AsiSI promoters group showed a stronger accumulation (Fig. 1F), possibly due to a more open chromatin conformation in these regions. This result suggests that promoters tend to recruit more DROSHA than gene body sites and intergenic sites, but also indicates that the presence of canonical pre-existing transcription is not a prerequisite for DROSHA association with damaged chromatin. To strengthen this second conclusion, we tested DROSHA recruitment at transcribed loci upon transcription inhibition. THZ1 (CDK7 inhibitor, CDK7i; Chipumuro et al., 2014), inhibits CDK7 kinase activity, and thus transcription initiation, and we have recently reported that by preventing dilncRNA transcription, THZ1 has the ability to reduce DDR foci formation in human cells (Pessina et al., 2019).

Indeed, THZ1 treatment prevented RNAPII C-terminal domain (CTD) phosphorylation (Pol IIO) (Fig. 1G) as well as 53BP1 foci formation (Fig. S1I) (Pessina et al., 2019). ChIP-qPCR at DSB-I and DSB-II, two AsiSI sites localized in transcribed regions, demonstrated that DROSHA is equally recruited in DIvA cells treated or not with THZ1 (Fig. 1H,I). This observation further indicates that DROSHA recruitment at DSBs does not require canonical gene transcription or *de novo* break-induced transcription of dilncRNA.

Recruitment of some DDR proteins to DNA lesions can be directly visualized by immunofluorescence in the form of microscopically detectable foci. Nevertheless, this is not possible for NHEJ repair factors whose association is limited to a few molecules in proximity to DNA ends. Since DROSHA displays a narrow accumulation at DSBs, we sought to assess its presence at an individual DSB, at single-cell level, by performing proximity ligation assays (PLAs; Söderberg et al., 2006). PLAs with antibodies against DROSHA and γH2AX generated several nuclear fluorescence signals upon cut formation, which disappeared following DROSHA knockdown, indicating that DROSHA is indeed recruited at γH2AX-positive, damaged chromatin (Fig. S1J,K). To strengthen these observations, and to demonstrate the close proximity between DROSHA and DSB DNA ends, we took advantage of a novel method recently developed in our laboratory, named ‘DNA damage *in situ* ligation followed by proximity ligation assay’ (DI-PLA) (Galbiati et al., 2017), in which a PLA is preceded by the ligation of a biotinylated oligonucleotide to DNA ends. In a DI-PLA, the signal derives from the proximity between antibodies against biotin (conjugated to the oligonucleotide) and any protein of interest, DROSHA in our case. By this approach, we observed a significant increase upon DNA damage of DROSHA–biotin DI-PLA signals, which were dramatically reduced upon DROSHA knockdown (Fig. S1L,M).

Altogether, genome-wide and individual site ChIP results, independently validated by PLA and DI-PLA assays, demonstrated that DROSHA is recruited to exposed DNA ends at DSBs. Importantly, this seems to occur regardless of the transcriptional status of the locus where DNA damage is induced.

### DROSHA recruitment to DSBs is part of the primary recruitment of DDR factors and is independent of ATM, DNA-PK and PARP1 activity

**Fig. 2.**
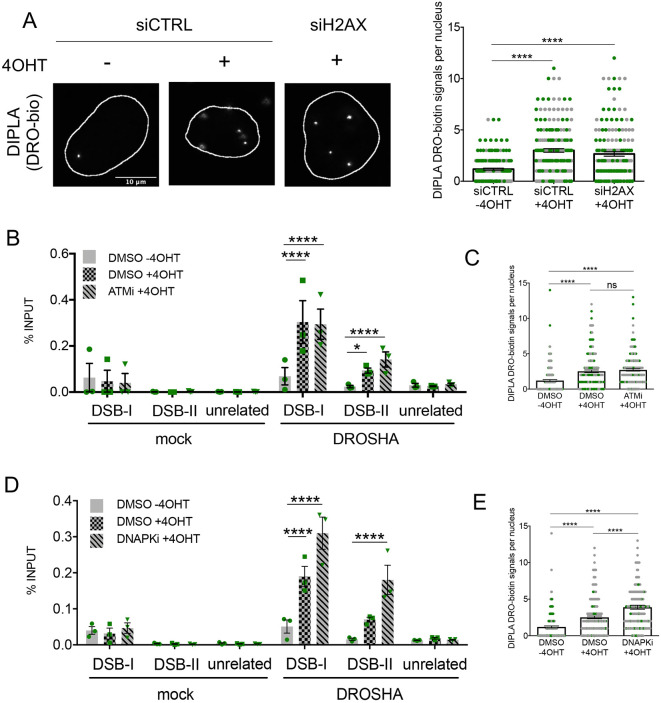
**DROSHA recruitment at DNA damage sites occurs independently of DDR signaling activation.** (A) Representative images of DI-PLA signal of DNA ends–DROSHA proximity in cut (+4OHT) and uncut (−4OHT) DIvA cells treated with control siRNA (siCTRL) and cut DIvA cells depleted of H2AX (siH2AX). The scatter plot represents the number of DI-PLA signals measured using CellProfiler automated software. Data are presented as mean±s.e.m. (200 cells, *n*=2). ****P≤0.0001 (one-way ANOVA with Tukey's multiple comparison test). (B) The bar plot shows the percentage of ChIP enrichment, relative to the input, of mock (no antibody) and DROSHA as detected by ChIP-qPCR in cut (+4OHT) and uncut (−4OHT) DIvA cells mock treated with DMSO and in cut DIvA cells treated with ATM inhibitor (ATMi), with primers matching DSB-I, DSB-II or an unrelated genomic region far from any annotated AsiSI sites. Data are mean±s.e.m. from three independent experiments. *P≤0.05; ****P≤0.0001 (two-way ANOVA with Tukey's multiple comparison test). (C) The scatter plot represents the number of DI-PLA signals measured using CellProfiler automated software in cut (+4OHT) and uncut (−4OHT) DIvA cells mock treated with DMSO and in cut DIvA cells treated with ATM inhibitor (ATMi). Data are presented as mean±s.e.m. (200 cells, *n*=2). ****P≤0.0001; ns, not significant (one-way ANOVA with Tukey's multiple comparison test). (D) The bar plot shows the percentage of ChIP enrichment, relative to the input, of mock (no antibody) and DROSHA as detected by ChIP-qPCR in cut (+4OHT) and uncut (−4OHT) DIvA cells mock treated with DMSO and in cut DIvA cells treated with DNA-PK inhibitor (DNA-PKi), with primers matching DSB-I, DSB-II or an unrelated genomic region far from any annotated AsiSI sites. Data are mean±s.e.m. from three independent experiments. ****P≤0.0001 (two-way ANOVA with Tukey's multiple comparison test). (E) The scatter plot represents the number of DI-PLA signals measured using CellProfiler automated software in cut (+4OHT) and uncut (−4OHT) DIvA cells mock treated with DMSO and in cut DIvA cells treated with DNA-PK inhibitor (DNA-PKi). Data are presented as mean±s.e.m. (200 cells, *n*=2). ****P≤0.0001 (one-way ANOVA with Tukey's multiple comparison test).

ATM and DNA-PK are two important kinases that modify chromatin at the initial steps of the DDR. In the chromatin surrounding a DSB, ATM-dependent H2AX phosphorylation is a requisite for the secondary recruitment of DDR mediators such as 53BP1 and MDC1, whereas it is dispensable for the primary recruitment of DDR sensors such as the MRN complex (Bekker-Jensen et al., 2006; Celeste et al., 2003). We have previously reported that DDRNAs generated by DROSHA and DICER are required, together with γH2AX, for the secondary recruitment of 53BP1 and MDC1 (Francia et al., 2016; Gioia et al., 2019; Pessina et al., 2019). Thus, we investigated whether DROSHA recruitment at DSBs was γH2AX-dependent. We addressed this point in DIvA cells knocked down for H2AX (Fig. S2A) by exploiting DI-PLA, an experimental approach that is not affected by γH2AX loss. We demonstrated that DROSHA is recruited to DNA lesions both in the presence or absence of H2AX (Fig. 2A; Fig. S2A). This suggests that H2AX is dispensable for DROSHA recruitment to DSBs.

To determine the role played by ATM, we performed ChIP-qPCR at AsiSI-induced DSBs upon treatment with KU60019 (ATMi), a molecule that specifically inhibits ATM kinase activity (Golding et al., 2009). In ATMi-treated DIvA cells, DROSHA occupancy at DSB-I and DSB-II was not affected (Fig. 2B) whereas, as expected, pATM and γH2AX accumulation was significantly reduced (Fig. S2B,C) (Caron et al., 2015). To further support this conclusion, we performed DI-PLA analysis in cells treated with ATMi, which confirmed DROSHA recruitment to DNA ends upon inhibition of ATM kinase activity (Fig. 2C; Fig. S2D). These results not only indicate that DROSHA recruitment is not controlled by ATM activation, but also strengthen the observation that DROSHA recruitment occurs independently of γH2AX formation.

Next, we tested the effect of DNA-PK inhibition by treating DIvA cells with NU7441 (DNAPKi), a specific inhibitor of DNA-PK kinase activity (Zhao et al., 2006) (Fig. S2E). Consistent with previous reports (Caron et al., 2015), ChIP-qPCR showed unaltered γH2AX levels upon DNA-PK inhibition (Fig. S2F). Interestingly, DROSHA recruitment to DSBs also was not impaired by the treatment, as detected by ChIP-qPCR (Fig. 2D) and DI-PLA analysis (Fig. 2E; Fig. S2G).

Finally, since some RNA-binding proteins transiently associate to DNA damage sites in a PARP1-dependent manner (Francica and Rottenberg, 2018), we tested whether DROSHA recruitment is controlled by this factor. However, DIvA cells treated with olaparib (PARPi) (Czyż et al., 2016), a molecule that inhibits the ability of PARP1 to synthesize poly(ADP-ribose) (PAR) chains (Fig. S2H), showed no significant changes in DROSHA accumulation at DSB-I and DSB-II (Fig. S2I,J).

Taken together, these results demonstrate that DROSHA is recruited to sites of DNA damage in a γH2AX-, ATM-, DNA-PK- and PARP 1-independent fashion.

### DROSHA recruitment to DSBs requires activity of the MRN complex, a DNA damage sensor

**Fig. 3.**
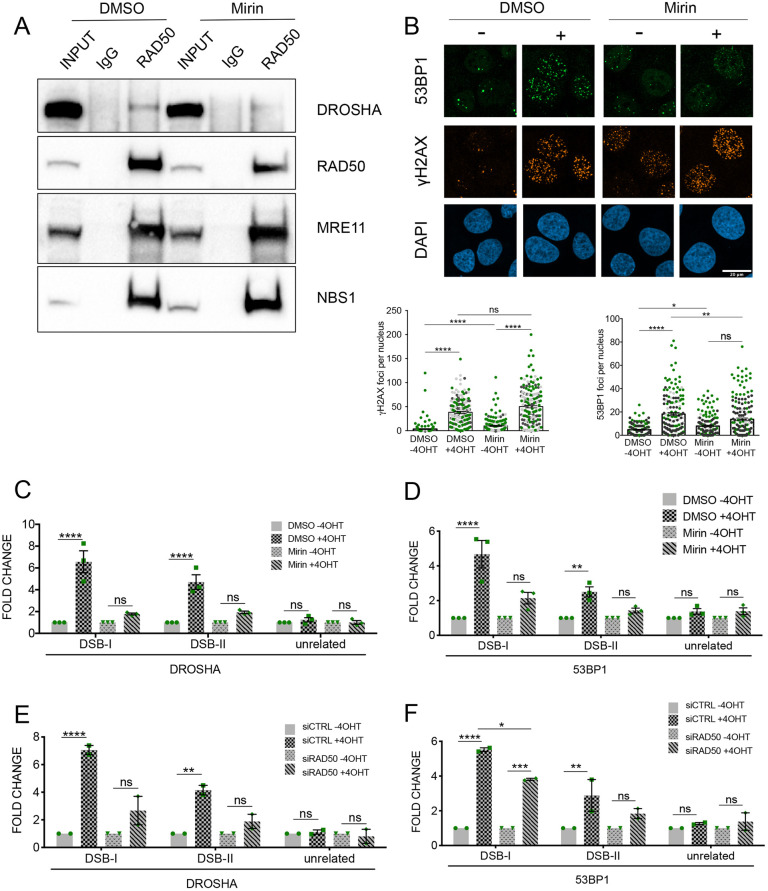
**The MRN complex DDR sensor supports DROSHA recruitment to DNA damage sites.** (A) Co-immunoprecipitation of RAD50 and DROSHA in 293T cells treated with 100 μM mirin or mock treated with DMSO. MRE11 and NBS1 blots are shown as controls for MRN complex co-immunoprecipitation. (B) Representative images of γH2AX and 53BP1 immunofluorescence in cut (+) and uncut (−) DIvA cells treated with 100 μM mirin or mock treated with DMSO. DNA is stained using DAPI. The scatter plots represent the number of γH2AX and 53BP1 foci in cut (+4OHT) and uncut (–4OHT) cells treated as indicated, measured using CellProfiler automated software. Data are presented as mean±s.e.m. (200 cells, *n*=3). *P≤0.05; **P≤0.01; ****P≤0.0001; ns, not significant (one-way ANOVA with Tukey's multiple comparison test). Scale bar: 20 μm. (C,D) The bar plots show the fold enrichment, relative to the uncut sample (−4OHT), of DROSHA (C) or 53BP1 (D) as detected by ChIP-qPCR in cut (+4OHT) and uncut (−4OHT) DIvA cells treated with mirin (100 μM) or mock treated with DMSO, with primers matching DSB-I, DSB-II or an unrelated genomic region far from any annotated AsiSI sites. Data are mean±s.e.m. from three independent experiments. **P≤0.01; ****P≤0.0001; ns, not significant (two-way ANOVA with multiple Tukey's comparison test). (E,F) The bar plots show the fold enrichment, relative to the uncut sample (−4OHT), of DROSHA (E) or 53BP1 (F) as detected by ChIP-qPCR in cut (+4OHT) and uncut (−4OHT) DIvA cells knocked down for RAD50 (siRAD50) or mock treated with non-targeting siRNA (siCTRL), with primers matching DSB-I, DSB-II or an unrelated genomic region far from any annotated AsiSI sites. Data are mean±s.e.m. from two independent experiments. *P≤0.05; **P≤0.01; ***P≤0.001; ****P≤0.0001; ns, not significant (two-way ANOVA with multiple Tukey's comparison test).

The MRN complex is recruited to sites of DNA damage independently of γH2AX and is required for dilncRNA biogenesis and DDR functions (Michelini et al., 2017). Thus, we tested whether the MRN complex also mediates DROSHA recruitment to DNA breaks. We initially performed RAD50 coimmunoprecipitation (co-IP) experiments in the presence of Benzonase to avoid DNA nucleic acid contamination and bridging among molecules. Excitingly, we observed that RAD50 interacts with DROSHA along with its canonical partners NBS1 and MRE11 (Fig. 3A). Mirin is an allosteric inhibitor of MRE11 that, by altering MRN structure, restricts MRN activities including MRE11 exonuclease activity, DNA binding and melting of DNA ends (Dupré et al., 2008; Jazayeri et al., 2008; Moiani et al., 2018; Shibata et al., 2014). We recently showed that mirin has the ability to prevent dilncRNA transcription in an *in vitro* assay (Sharma et al., 2021). Interestingly, we observed that DROSHA–RAD50 interaction is reduced when cells are treated with mirin, while the association between RAD50, NBS1 and MRE11 remained unaffected (Fig. 3A). These results demonstrate an unanticipated interaction between DROSHA and the MRN complex, which can be impaired by binding of the MRN allosteric inhibitor mirin.

To test whether MRN activity also controls DROSHA localization at DSBs, we performed ChIP-qPCR in DIvA cells treated or not with mirin. Because MRN is required for DNA repair, mirin treatment resulted in an increased background level of damage as detected by γH2AX immunofluorescence (Fig. 3B). Thus, to evaluate how mirin affects the accumulation of DDR factors upon DSB generation, we calculated the ratio between the signals detected in damaged and undamaged samples for each condition. Excitingly, upon mirin treatment both DSB-I and DSB-II displayed reduced DROSHA enrichment (Fig. 3C) despite exhibiting unaltered γH2AX levels (Fig. S3A). As expected, mirin treatment strongly reduced pATM induction (Fig. S3B). Importantly, we also observed, by ChIP-qPCR and immunofluorescence, that 53BP1 accumulation at the site of damage was significantly reduced in DIvA cells treated with mirin (Fig. 3B,D), a result in line with previous reports (Francia et al., 2016, 2012). Importantly, DROSHA recruitment at DSB-I and DSB-II was also impaired in DIvA cells in which the MRN complex was destabilized by RAD50 knockdown (Fig. 3E; Fig. S3C,D). Again, as a consequence of loss of DROSHA recruitment, 53BP1 accumulation at the same site was significantly reduced (Fig. 3F).

These results suggest that the MRN complex and its ‘DNA-bridging’ activity inhibited by mirin is key in controlling DROSHA recruitment to break sites, which in turn promotes the secondary recruitment of the DDR mediator 53BP1. In conclusion, DROSHA is recruited at DSBs by the MRN complex, likely by protein–protein interaction and independently of RNAP II-mediated *de novo* transcription occurring at damaged sites.

### A role for DROSHA in NHEJ repair

**Fig. 4.**
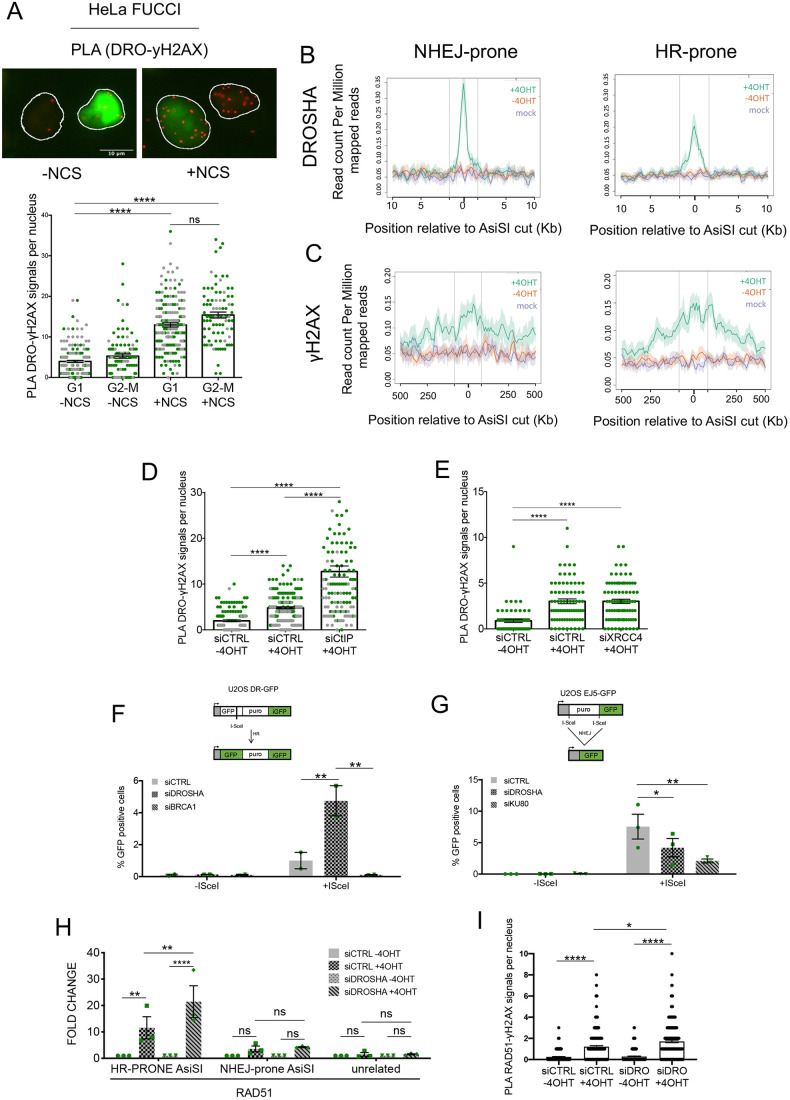
**DROSHA recruitment to DSBs occurs throughout the cell cycle and preferentially at NHEJ-prone DSBs.** (A) The FUCCI (fluorescence ubiquitination cell cycle indicator) cell cycle sensor consists of a fluorescent protein-based system that employs both a red (RFP) and a green (GFP) fluorescent protein fused to different regulators of the cell cycle: CDT1 and geminin. During the cell cycle, these two proteins display temporal regulation that results in the biphasic cycling their levels during the cell cycle. In the G1 phase of the cell cycle only CDT1 tagged with RFP is present and appears as red fluorescence within the nuclei. In the S, G2 and M phases only geminin tagged with GFP remains, resulting in cells with green fluorescent nuclei. Representative images of PLA signal (red puncta) of γH2AX–DROSHA proximity in NCS treated (12 ng/ml) and untreated HeLa FUCCI cells. Nuclei are outlined in white. The scatter plot shows the number of PLA signals for cells in the indicated phases of the cell cycle, as measured using CellProfiler automated software. Data are presented as mean±s.e.m. (250 cells, *n*=2). ****P≤0.0001; ns, not significant (one-way ANOVA with Tukey's multiple comparison test). (B,C) Mean±s.e.m. DROSHA (B) or γH2AX (C) ChIP-seq signals of NHEJ-prone (left) or HR-prone (right) AsiSI sites, over 1 Mb or 20 kb windows, respectively, and centered at the AsiSI site, are shown for cut (+4OHT, green), uncut (−4OHT, red) or mock (magenta) samples. All the AsiSI sites assayed are included in the most cut AsiSI sites (Iannelli et al., 2017). Vertical lines indicate the boundaries of the region used to center all the top 50 AsiSI sites. (D) The scatter plot represents the number of γH2AX–DROSHA PLA signals measured using CellProfiler automated software in cut (+4OHT) and uncut (−4OHT) DIvA cells mock treated with non-targeting siRNA (siCTRL) and in cut DIvA cells knocked down for CtIP (siCtIP). Data are presented as mean±s.e.m. (150 cells, *n*=2). ****P≤0.0001 (one-way ANOVA with Tukey's multiple comparison test). (E) The scatter plot represents the number of γH2AX–DROSHA PLA signals measured using CellProfiler automated software in cut (+4OHT) and uncut (−4OHT) siCTRL-treated DIvA cells and in cut DIvA cells knocked down for XRCC4 (siXRCC4). Data are presented as mean±s.e.m. (90 cells, *n*=1). ****P≤0.0001 (one-way ANOVA with Tukey's multiple comparison test). (F) Schematic of the DR-GFP reporter used to monitor HR in U2OS cells. Puro, puromycin resistance gene; iGFP, internal GFP repeat. U2OS DR-GFP cells were transfected with control siRNA (siCTRL), siRNA against DROSHA (siDROSHA) or siRNA against BRCA1 (siBRCA1). Cells were induced with doxycycline (5 μg/ml; +ISceI) or mock treated with DMSO (–ISceI) 48 h before analysis. GFP-positive cells were analysed by flow cytometry to score the HR repair efficiency. The bar plot shows mean±s.e.m. percentage of GFP-positive cells from two independent experiments. **P≤0.01 (two-way ANOVA with Šidák correction). (G) Schematic of the EJ5-GFP reporter used to monitor NHEJ in U2OS cells. U2OS EJ5 cells were transfected with control siRNA (siCTRL), siRNA against DROSHA (siDROSHA) or siRNA against KU80 (siKU80). Cells were transfected with ISceI (+ISceI) or empty vector plasmids (−ISceI) 48 h before analysis. GFP-positive cells were analysed by flow cytometry to score the NHEJ repair efficiency. The bar plot shows the mean±s.e.m. percentage of GFP-positive cells from three independent experiments. *P≤0.05; **P≤0.01 (two-way ANOVA with Šidák correction). (H) The bar plot shows the fold change in ChIP enrichment, relative to the uncut sample (−4OHT), of RAD51 as detected by ChIP-qPCR in cut (+4OHT) and uncut (−4OHT) DIvA cells knocked down for DROSHA (siDROSHA) or mock treated with non-targeting siRNA (siCTRL), with primers matching an NHEJ-prone ASiSI site, an HR-prone AsiSI site or an unrelated genomic region far from any annotated AsiSI sites. Data are presented as mean±s.e.m. of three independent experiments. **P≤0.01; ****P≤0.0001; ns, not significant (two-way ANOVA with Tukey's multiple comparison test). (I) The scatter plot represents the number of RAD51–γH2AX PLA signals measured using CellProfiler automated software in cut (+4OHT) and uncut (−4OHT) DIvA cells either knocked down for DROSHA (siDRO) or mock treated with non-targeting siRNA (siCTRL). Data are presented as the mean±s.e.m. (250 cells, *n*=3). *P≤0.05; ****P≤0.0001 (one-way ANOVA with Tukey's multiple comparison test).

A role for DROSHA in mediating DNA–RNA hybrid formation at DSBs has been proposed in relation to DNA repair by both NHEJ and HR (Lu et al., 2018). However, the role played by DROSHA in DNA repair remains in large part unclear. Whereas NHEJ is active across the entire cell cycle, HR is restricted to S and G2 phases (Hustedt and Durocher, 2016). To investigate whether DROSHA recruitment occurs throughout the cell cycle or in S and G2 phases only, we performed PLA analyses of γH2AX and DROSHA in the FUCCI cellular system (Goto et al., 2015), a well-established fluorescent-protein-based system that allows a visual readout of the cell cycle phase of each cell at a given moment. Treatment with the radiomimetic drug neocarzinostatin (NCS) induced a significant increase of PLA signals that did not show significant differences throughout the cell cycle (Fig. 4A). This observation indicates that DROSHA, similarly to NHEJ factors, operates at DSBs in any cell cycle phase.

A previous study (Aymard et al., 2014) in the DIvA cellular system reported that the NHEJ factor XRCC4 associates with all AsiSI-induced DSBs in both G1 and S-G2 phases, whereas the HR factor RAD51 associates only to a number of AsiSI-induced DSBs more strongly in G2 phase. This allows the distinction between two subsets of AsiSI-induced DSBs, referred to as NHEJ-prone and HR-prone sites (Aymard et al., 2014). On the basis of this classification, we separated our set of top 50 AsiSI-induced DSBs into NHEJ- and HR-prone sites and, using ChIP-seq analyses, we observed that DROSHA occupancy was higher in NHEJ-prone DSB sites relative to occupancy at HR-prone sites (Fig. 4B; Fig. S4A). This occurs despite the two sets of sites exhibiting comparable γH2AX ChIP-seq signals (Fig. 4C; Fig. S4B).

To strengthen this observation and test the hypothesis that DROSHA is preferentially involved in NHEJ repair, we suppressed HR to boost NHEJ repair pathway usage and analyzed DROSHA association to DSBs using a PLA (Yun and Hiom, 2009). Excitingly, knockdown of CtIP (also known as RBBP8), an essential HR factor involved in DNA end resection (Fig. S4C), strongly enhanced the number of γH2AX–DROSHA PLA signals, suggesting that HR blockage indeed increases the recruitment of DROSHA to site of damage (Fig. 4D; Fig. S4D). Importantly, the number of γH2AX foci did not increase upon CtIP silencing (Fig. S4E) ruling out the possibility that the augmented PLA signal depended on a global increase of DNA damage. Conversely, XRCC4 knockdown (Fig. S4F), a cofactor of ligase IV acting in the final steps of NHEJ (Chang et al., 2017), did not significantly alter γH2AX–DROSHA PLA signals (Fig. 4E; Fig. S4G). Taken together, these observations reveal a stronger affinity of DROSHA for DSBs that preferentially undergo NHEJ repair.

Finally, to obtain a functional readout of DROSHA inactivation on these two different repair pathways, we took advantage of two well-established reporter cellular systems: U2OS DR-GFP (specific for HR; Fig. 4F) and U2OS EJ5-GFP (specific for NHEJ; Fig. 4G). In both systems, the successful repair of a site-specific DSB generated by I-SceI meganuclease results in the restoration of a functional GFP gene that can be accurately detected by fluorescence-activated cell sorting (FACS) analyses. In DR-GFP cells, consistent with the increased DROSHA recruitment to DSBs observed upon CtIP silencing, HR frequency was strongly enhanced upon DROSHA inactivation (Fig. 4F; Fig. S4H). As expected, BRCA1 knockdown resulted in a dramatic reduction in HR efficiency (Fig. 4F; Fig. S4H). Importantly, we assessed cell cycle distribution in each individual experiment, and we observed that it was never significantly altered (Fig. S4I). In the U2OS EJ5 cells, we observed that the number of GFP-positive events was significantly reduced upon DROSHA inactivation, as well as upon KU80 knockdown used as control (Fig. 4G; Fig. S4J). Moreover, in this cell line, cell cycle distribution was not altered by DROSHA inactivation (Fig. S4K). Taken together, these results indicate that DROSHA, by controlling 53BP1 recruitment (Francia et al., 2016, 2012), promotes NHEJ, thus inhibiting HR.

To further corroborate this hypothesis, we tested whether DROSHA knockdown could stimulate the recruitment of HR factors at DSBs. Thus, we performed ChIP-qPCR analysis with an antibody against RAD51 in DIvA cells knocked down for DROSHA, focusing our attention on an HR-prone site and on an NHEJ-prone site, as reported previously (Aymard et al., 2014). The sites displayed a comparable cut efficiency (Fig. S4L) and, in accordance with our findings in the DR-GFP HR reporter system, we observed that DROSHA inactivation stimulates RAD51 recruitment at the HR-prone site, whereas it does not affect basal levels of RAD51 recruitment at the NHEJ-prone site (Fig. 4H). Similarly, RAD51–γH2AX PLA signals per nucleus were increased in DROSHA-knockdown DIvA cells (Fig. 4I; Fig. S4M), confirming that DROSHA counteracts recruitment of HR factors at DSBs.

Taken together, these results demonstrate an unanticipated role for DROSHA at sites of DNA damage to promote DSB repair by NHEJ.

## DISCUSSION

Previously, our group and others demonstrated that DNA damage sites induce the synthesis of non-coding transcripts, which can be further processed into smaller RNAs by components of the RNA interference machinery and together play an important role in DDR signaling (Francia et al., 2012; Michalik et al., 2012; Michelini et al., 2017; Wei et al., 2012). We recently reported that RNAP II is recruited in a MRN-dependent manner to DSBs occurring both at repetitive and unique chromosomal loci. Once there, RNAP II synthesizes dilncRNAs bidirectionally, from and towards DNA ends, favoring protein–RNA interactions that promote liquid–liquid phase separation of DDR factors at DNA damage sites (Pessina et al., 2019). Induction of dilncRNA has been observed in several distinct cell systems, including U2OS DIvA cells (Michelini et al., 2017). Nevertheless, whether the primary transcript is processed locally at the site of DNA damage or elsewhere remained unknown. DICER, generally considered exclusively cytoplasmic (Much et al., 2016), was recently shown to be recruited to sites of DNA damage in a phosphorylated form (Burger and Gullerova, 2018; Burger et al., 2017).

Here we report, for the first time on a broad scale, that DROSHA accumulates at sites of DNA damage occurring in different positions across the genome. Our results indicate that DROSHA is recruited to DSBs via interaction with the DNA damage sensor MRN but independently of canonical gene and dilncRNA transcription. Importantly, this recruitment mechanism is not different from that described for the recruitment of DROSHA during microRNA biogenesis and at gene promoters, which occurs via protein–protein interaction and not via direct binding with RNA (Gregory et al., 2004; Gromak et al., 2013).

It should be noted that at all DSBs investigated, DROSHA binding was absent in unperturbed conditions (prior to damage generation), demonstrating that we are describing events that are strictly dependent on acute DNA damage generation. DROSHA recruitment occurred at DSBs generated in different genomic contexts; however, its presence was more evident at DSBs falling in promoters of active genes, possibly due to the presence of a more open chromatin. Similar to recruitment of the MRN complex, DROSHA recruitment did not depend on H2AX, and so belongs to the group of DDR factors directly recruited at lesions as primary sensors. Importantly, DROSHA recruitment was also independent from the kinase activity of ATM and DNA-PK, suggesting that DROSHA recruitment to DSBs is an early event during DDR activation.

The MRN complex performs several important activities at DSBs: it not only acts as a DNA damage sensor and recruits RNAP II transcriptional machinery to activate the DDR, but also controls DNA end resection initiation in S-G2 phase and favours DNA ‘melting’, a transient unwinding of the DNA double helix (Sharma et al. 2021; Paull, 2015; Stracker and Petrini, 2011). Intriguingly, DROSHA–MRN interaction was reduced upon treatment with the MRE11 allosteric inhibitor mirin, suggesting that the conformational change induced by binding to this small molecule has the ability to alter the affinity of the MRN complex for protein–protein interactions. Indeed, DROSHA recruitment to DSBs was reduced in cells treated with mirin or depleted of RAD50, demonstrating that the DROSHA–MRN interaction controls DROSHA association with DNA damage sites.

Despite the fact that DROSHA can interact with the RNAP II CTD at specific gene promoters (Gromak et al., 2013), we observed that DROSHA recruitment to DSBs was not promoted by RNAP II transcription, as demonstrated by transient inhibition of RNAP II using a CDK7 inhibitor. This result confirms that DROSHA acts in association with the DNA damage sensor MRN instead of being recruited by RNA molecules. Importantly, this evidence offers a likely explanation of why DROSHA is not recruited to any gene promoter and is instead recruited specifically following DNA damage. Once at the site of a break, DROSHA processes dilncRNA into DDRNAs, thus promoting 53BP1 recruitment. Indeed, DROSHA inactivation results in increased levels of dilncRNA (Michelini et al., 2017).

We recently have shown that dilncRNA form hybrid structures with single-stranded DNA at resected DNA ends (D'Alessandro et al., 2018), and others have shown that DROSHA promotes DNA–RNA hybrid formation (Lu et al., 2018). Nevertheless, it is not easy to imagine how an endoribouclease could support the formation of this structure. One possibility is that dilncRNA processing by DROSHA contributes to dilncRNA transcription termination, thus allowing the engagement of a partially processed RNA in DNA–RNA hybrid formation.

DROSHA recruitment is restricted to the proximity of DNA ends, a localization that very much resembles the one of NHEJ repair factors such as XRCC4 (Aymard et al., 2014). Consistently, DROSHA accumulates preferentially at NHEJ-prone sites and its association with damaged chromatin is enhanced by HR suppression and NHEJ stimulation. Indeed, DROSHA knockdown strongly impairs NHEJ repair efficiency to an extent not very different from that caused by the inactivation of KU80, a central player in this repair pathway.

The observation that DROSHA is dispensable for HR is in contrast with a recent report that shows instead that DROSHA controls both HR and NHEJ (Lu et al., 2018). Possible explanations for this discrepancy may include altered cell cycle distribution, which we occasionally observed in some cell lines upon DROSHA knockdown using siRNA pools. In our hands, this specifically occurred in DR-GFP cells when using some old generation siRNAs, possibly due to off-target effects. For this reason, we carefully confirmed that cell cycle progression remained unaffected in all the experiments included in this study.

DDRNAs with telomeric sequence are produced at uncapped telomeres, a condition that elicits a full DDR response (Rossiello et al., 2017). Importantly, DROSHA inactivation reduces fusions at uncapped telomeres, a NHEJ DNA repair event.

Overall, it is becoming increasingly evident that DICER and DROSHA are essential for DDR activation and are involved in DNA repair (d'Adda di Fagagna, 2014; Francia, 2015), a function that might contribute to the prevention of cancer development. Somatic mutations of DROSHA have been shown to be frequent and underlie high risk of Wilms' tumors (Spreafico et al., 2016; Torrezan et al., 2014; Wegert et al., 2015), and DROSHA depletion has been reported to be implicated in the promotion of a migratory phenotype in lung cancer cells (Frixa et al., 2017). Moreover, expression of DROSHA has been recently shown to be impaired in breast cancer patients, possibly in the attempt to stimulate HR, although the molecular mechanisms by which this may be relevant for cancer development remain to be defined (Poursadegh Zonouzi et al., 2017). Finally, it is important to mention that NHEJ repair events are the most frequently occurring in our body, especially in terminally differentiated cells such as neurons, with important implications for the understanding of the molecular mechanism behind neurodegeneration. Indeed DROSHA is involved in the cellular response to DNA damage in neuronal cells carrying a Parkinson’s disease-linked mitochondrial mutation (Pignataro et al., 2017).

In conclusion, we have characterized the mechanism behind DROSHA recruitment to DNA damage sites and have identified an unanticipated role for DROSHA in inhibiting HR and promoting the NHEJ DNA repair pathway.

## MATERIALS AND METHODS

### Cell culture and treatments

DIvA cells (AsiSI-ER-U20S; a gift from Dr Gaëlle Legube, Molecular, Cellular and Developmental Biology Unit, Centre de Biologie Intégrative, UPS, CNRS, Toulouse, France; Iacovoni et al., 2010) were cultured in Dulbecco's modified Eagle's medium (DMEM) (Life Technologies) without Phenol Red supplemented with 10% fetal bovine serum (FBS; Euroclone), 1% L-glutamine (Euroclone), 1% pyruvate (Microtech), 2.5% HEPES (Euroclone) and 1% penicillin-streptomycin (Euroclone). Cells were selected with puromycin (1 μg/ml). For AsiSI- dependent DSBs induction, cells were treated with 300 nM 4OHT (Sigma-Aldrich) for 4 h. HeLa-FUCCI cells were grown in DMEM (Euroclone), supplemented with 10% FBS (Euroclone), 1% L-glutamine and 1% penicillin-streptomycin. DNA damage was induced using the radiomimetic drug neocarzinostatin (NCS) (Sigma-Aldrich) at a concentration of 12 ng/ml for 20 min at 37°C. 293T cells were grown in DMEM (Euroclone), supplemented with 10% FBS (Euroclone) and 1% L-glutamine. All the cell lines were grown at 37°C under a humidified atmosphere with 5% CO_2_. To inhibit ATM or DNA-PKcs activity, KU60019 (Sigma-Aldrich) or NU7441 (Tocris Bioscience) were added to the medium at the final concentrations of 10 μM and 2 μM, respectively, 1 h before the addition of 4OHT. To inhibit CDK7, THZ1 (APExBIO) was added to the medium at a final concentration of 1 μM for the last 3 h of 4OHT-mediated induction. To inhibit MRN activity, mirin (Sigma-Aldrich) was added to the medium at a final concentration of 100 μM 30 min before the addition of 4OHT. To inhibit PARP1, olaparib was added to the medium at a final concentration of 1 μM 1 h before the addition of 4OHT.

### Antibodies

For complete list of antibodies used see Table S1. The anti-MRE11 antibody was a gift from S. P. Jackson (Wellcome Trust/Cancer Research UK Gurdon Institute, Cambridge, UK) and has been previously reported by Francia et al. (2012), Michelini et al. (2017) and Pessina et al. (2019). The anti-KU80 antibody used has been previously reported by Sharma et al. (2021).

### Chromatin immunoprecipitation

Cells were cross-linked for 5.5 min at room temperature with fixation buffer (1% formaldehyde, 100 mM NaCl, 1 mM EDTA, 0.5 mM EGTA and 50 mM HEPES pH 7.4). Cross-linking was quenched by addition of glycine (125 mM). Fixed cells were rinsed twice in 1× phosphate-buffered saline (PBS), collected by scraping and centrifuged at 300 ***g*** for 5 min at 4°C. Pellets were resuspended in cold B1 buffer [0.25% Triton X-100, 1 mM EDTA, 0.5 mM EGTA, 10 mM Tris-HCl pH 8, protease inhibitors (Roche) and microcystin (Enzo Life Sciences)] by mixing for 10 min on a rotating wheel at 4°C and then centrifuged at 300 ***g*** for 5 min at 4°C. The same steps were repeated with cold buffer B2 (200 mM NaCl, 1 mM EDTA, 0.5 mM EGTA, 10 mM Tris-HCl pH 8, protease inhibitors and microcystin). Finally, pellets were re-suspended in cold buffer B3 (1×TE with 0.5 mM EGTA) in a suitable volume. Pellets were sonicated using a focused-ultrasonicator (Covaris; duty, 5.0; PIP, 140; cycles, 200; amplitude, 0; velocity, 0; dwell, 0; microTUBEs with AFA fiber). Sonicated chromatin was diluted in RIPA buffer (1% Triton X-100, 0.1% Na deoxycholate, 0.1% SDS, 64 mM NaCl and 10 mM Tris-HCl pH 8.0) to give a concentration of approximately 100 μg in 400 μl per ChIP. Samples were pre-cleared for 2 h, rotating at 4°C, with 20 μl of magnetic beads (Dynabeads Protein G, Life Technologies) per ChIP. Samples were then incubated overnight rotating at 4°C with specific antibodies (Table S1) or no antibody (mock). The bound material was recovered by 2 h incubation with 20 μl of magnetic beads per ChIP. Beads were then washed, rotating at 4°C for 10 min, four times in RIPA buffer, once in LiCl buffer (250 mM LiCl, 0.5% NP-40, 0.5% Na deoxycholate, 1 mM EDTA and 10 mM Tris-HCl pH 8) and finally in 1× TE. ChIPed material was eluted by 15 min incubation at 65°C with 100 μl elution buffer (1% SDS, 10 mM EDTA and 50 mM Tris-HCl pH 8). Samples were reverse-crosslinked by incubation with proteinase K (Invitrogen) at 37°C for 5 h and then at 65°C overnight. DNA was cleaned up by QIAquick PCR purification column (Qiagen), according to the manufacturer's instructions, and eluted in 30 μl of elution buffer (EB).

### ChIP-seq data analysis

The purified ChIPed DNA was sent to IGA (Institute of Applied Genomics, Udine), which performed quality and quantity assessment, library preparation and sequencing using standard Illumina TruSeq for HiSeq 2000 reagents. Each sample was sequenced in single-end (50 bp) mode for a total of 40 million reads per sample. Preliminary sequencing quality assessment was performed using FastQC (http://www.bioinformatics.babraham.ac.uk/projects/fastqc/). The samples passing the literature quality standards (Phred quality score >30) were aligned on the human genome (GRCh37/hg19) using BWA (Li and Durbin, 2009) with default parameters. In order to maintain the collinearity between the read signal and the protein occupancy on the genome, multiple-matching reads were eliminated using ad-hoc SAMtools (Li et al., 2009) and UNIX shell integrated scripts. Subsequently, peak calling was performed with MACS 1.4.2 (Zhang et al., 2008). Preliminarily, a parameter evaluation step was performed in order to converge on the optimal parameters for the peak calling. Main parameters in the MACS algorithm are the MFOLD (enriched quality interval) and the bandwidth (shifting model length). Automatic MACS runs were submitted incrementing both parameters in order to select the most reliable values according to the peak discovery rate. This evaluation was carried out using two different proteins characterized by different peak shapes (γH2AX and XRCC4). After that, all the proteins underwent peak calling. The output was intersected with the AsiSI site database using BEDtools (Quinlan and Hall, 2010). In the end, quantitative analysis of induced (+4OHT) and uninduced (−4OHT) dataset was carried out via PscanChIP (Zambelli et al., 2013) using default parameters. In particular, we focused on a proximal region surrounding the AsiSI site by empirically tailoring the frame of the algorithm on the peak length coming from MACS. Moreover, PscanChIP produced a final list of AsiSI sites ranked for the imbalance of γH2AX signal via χ^2^ test. Finally, data visualization was obtained using the ngs.plot package, an R based data mining and visualization tool for next-generation sequencing data (Shen et al., 2014). This tool is based on two steps of normalization. In the first step of length normalization, regions of variable sizes are equalized. In the second step, the vectors are normalized against the corresponding library size to generate the so-called reads per million mapped reads (RPM) values that allow two NGS samples to be compared regardless of differences in sequencing depth.

### qPCR analysis

Equal volumes of immunoprecipitated chromatin were used for standard qPCR on a region proximal to the DSB-I or DSB-II sites, or on a region far from any annotated AsiSI site as control, as described previously (Iacovoni et al., 2010). Values for each immunoprecipitated sample were normalized to their inputs. qPCR was performed using QuantiTect SYBR Green PCR Master Mix (QIAGEN) on a Roche LightCycler 480 or LightCycler 96. For the experiments shown in Fig. 3D–F and Fig. 4H, the PCR program used consisted of one cycle of denaturation (95°C, 5 min); 45 cycles of denaturation (95°C, 10 s), annealing (60°C, 10 s) and extension (72°C, 10 s); and one cycle of a melting curve (60°C–90°C). For the remaining experiments, the PCR program was the following: one cycle of denaturation (95°C, 15 min); 50 cycles of denaturation (95°C, 15 s), annealing (60°C, 20 s) and extension (72°C, 30 s); and one cycle of a melting curve (60°C–90°C). For a complete list of primers used see Table S2.

### RNA interference

ON-TARGET plus siRNA oligonucleotides (Dharmacon) were transfected at a final concentration of 10 nM using Lipofectamine RNAiMax (Life Technologies) following the manufacturer's protocol. 72 h later, DNA damage was induced and samples were collected. The sequences (5′ to 3′ orientation) of the siRNA oligonucleotides used are reported in Table S3.

### Indirect immunofluorescence and imaging analysis

Cells were grown on coverslips, fixed in 4% paraformaldehyde for 10 min at room temperature and permeabilized in Triton X-100 (0.2% in 1× PBS) for 10 min at room temperature. After two washes in 1× PBS, coverslips were blocked in 1× PBG (10× stock PBG: 5% BSA and 2% gelatin from cold water fish skin in PBS) for 1 h at room temperature. Primary antibody (Table S1) incubation was performed for 1 h at room temperature in a humid chamber. After three washes in 1× PBG, secondary antibody incubation was performed for 1 h at room temperature in a humid chamber. After two washes in 1× PBG and two washes in 1× PBS, incubation with DAPI 0.2 μg/ml (Sigma-Aldrich) was performed for 2 min at room temperature. After two washes in 1× PBS and one wash in deionized water, coverslips were mounted onto glass slides using Aqua Poly/Mount (Polysciences) and allowed to dry overnight at room temperature. Acquisitions were carried out with a wide-field epifluorescence microscope (Olympus IX71) equipped with PlanApo 60×/1.40NA oil immersion objective, a Cool SNAP ES camera (Photometrics) and driven by MetaMorph software (Universal Imaging Corporation).

Moreover, some immunofluorescence images were acquired using a confocal laser-scanning microscope (Zeiss LSM 800) equipped with four lasers [diode laser 405 nm (5 mW), diode laser 488 nm (10 mW), diode laser 561 nm (10 mW) and diode laser 640 nm (5 mW)], two Master gain with high sensitivity and a 63×/1.4NA objective. The LSM 800 was used to capture 10–18 *z*-sections (230 nm) with Zeiss Zen Blue 2.6 software. Comparative immunofluorescence analyses were performed using the automated image analysis software CellProfiler 2.1.1 (Carpenter et al., 2006).

### *In situ* proximity ligation assay

The Duolink In Situ Orange Starter Kit Mouse/Rabbit (Sigma-Aldrich) was used according to the manufacturer's protocol. Briefly, cells were fixed, permeabilized and blocked as described above for immunofluorescence studies. Then, cells were incubated with primary antibodies diluted in 1× PBG for 1 h at room temperature (Table S1). The cells were washed 3× in 1× PBG and incubated with the PLA probes (secondary antibodies conjugated with oligonucleotides) for 1 h at 37°C in a humid chamber. Cells were washed twice in buffer A (supplied with the kit) and the ligation reaction was carried out at 37°C for 30 min in a humid chamber followed by a wash in buffer A. The cells were then incubated with the amplification mix for 1.5 h at 37°C in a darkened humidified chamber. After washing with buffer B (supplied with the kit), followed by a 1 min wash with 0.01× buffer B, the cells were incubated with DAPI 0.2 μg/ml (Sigma-Aldrich) and mounted. Images were acquired using a wide-field epifluorescence microscope (Olympus IX71) equipped with a 60× objective. Quantification of nuclear PLA dots was performed using the automated image analysis software CellProfiler 2.1.1 (Carpenter et al., 2006).

### DNA damage *in situ* ligation proximity ligation assay

After fixation and permeabilization as described above for immunofluorescence, cells were treated for DI-PLA. Coverslips were washed twice for 5 min in 1× CutSmart buffer (NEB) and once in 1× blunting buffer (NEB). Afterwards, blunting was performed at room temperature for 60 min in a final volume of 50 μl for each coverslip [38.5 μl H_2_O, 5 μl 10× blunting buffer (NEB), 5 μl dNTP 1 mM (NEB), 0.5 μl BSA (molecular biology grade, 20 mg/ml; NEB) and 1 μl blunting enzyme mix (NEB)]. Coverslips were then washed twice in 1× CutSmart buffer and twice in 1× T4 ligase buffer (NEB). Then, *in situ* ligation was performed overnight at 16°C in a sealed humid chamber, in 100 μl final volume per coverslip using: 2 μl T4 Ligase (NEB), 5 μl 10 μM biotinylated linker (5′-TACTACCTCGAGAGTTACGCTAGGGATAACAGGGTAATATAGTTT[biotin–dT]TTTCTATATTACCCTGTTATCCCTAGCGTAACTCTCGAGGTAGTA-3′), 10 μl 10× T4 Ligase Buffer (NEB), 1 μl dATP solution 100 mM (NEB), 1 μl BSA (molecular biology grade, 20 mg/ml; NEB) and 81 μl H_2_O. Coverslips were washed twice in PBS and processed as described for PLA using a primary antibody against biotin partnered with a primary antibody directed against the protein under investigation (Table S1; Galbiati et al., 2017).

### Immunoblotting

Cells were lysed in Laemmli sample buffer [2% SDS, 5% glycerol, 1.5% dithiothreitol (DTT), 0.01% Bromophenol Blue and 60 mM Tris-HCl pH 6.8]. Collected cells were sonicated (Diagenode) with three bursts of 15 s and heated for 10 min at 95°C. 10–15 μl of lysate was loaded on an SDS–polyacrylamide gel with a width of 1 mm, along with 7 μl of molecular weight markers (Bio-Rad). A voltage of 60 V for the stacking gel and 150 V for the resolving gel were applied. Gels were run in Tris-glycine electrophoresis buffer (25 mM Tris, 250 mM glycine and 0.1% SDS). For western blotting analysis, proteins were transferred to a 0.2 μm nitrocellulose membrane using the Trans-Blot Turbo Transfer System apparatus (Bio-Rad). The transfer was performed at 25 V for 7 or 10 min (according to the molecular weight of the proteins under investigation). Membranes were incubated with 5% skim milk in TBS-T buffer (TBS containing 0.1% Tween-20) for 1 h, followed by overnight incubation at 4°C with primary antibody and three washes with TBS-T before 1 h incubation at room temperature with the specific HRP-conjugated secondary antibody. Chemiluminescence detection was done by incubation with Luminata Classico or Crescendo (Millipore). Proteins were visualized by autoradiography on ECL films (Amersham) using various exposure times and manual development, or by using a Chemidoc imaging system (Bio-Rad).

### Immunoprecipitation

HEK293T cells treated with mirin (100 μM) or mock treated with DMSO for 2.5 h, were collected and washed in 1× TBS (ice-cold) and resuspended in lysis buffer [50 mM Tris-HCl pH 7.5, 150 mM NaCl, 0.5% NP-40, 5 mM MgCl_2_, 5% glycerol, 1× protease inhibitors (Roche) and 1× phosphatase inhibitors tablet (Roche)] supplemented with 1 μl/ml Benzonase (250 units/ml, Sigma). Lysates were incubated at 4°C for 45 min. Lysates were then cleared, and an equal amount of total protein extract was used for each immunoprecipitation. Primary antibody, pre-incubated with Protein G Dynabeads (Invitrogen), was added and left at 4°C on a wheel for a further 2 h. The beads were gently collected using a magnetic rack (Invitrogen), washed three times with 1× lysis buffer and resuspended in 50 μl of sample loading buffer (Invitrogen).

### Cell cycle analyses by FACS

U2OS cells were collected 72 h post transfection with siRNA, washed in PBS and fixed in 75% ethanol overnight at 4°C. A total of 10^6^ fixed cells for each condition were washed once in PBS containing 1% BSA and resuspended in PBS containing propidium iodide (50 μg/ml) and RNaseA (250 μg/ml), then incubated overnight in the dark. FACS analysis was performed on single-cell suspensions. For each measurement, at least 10,000 cells were acquired. Samples were acquired either on a FACSCanto II (Becton Dickinson) or a Bio-Rad S3e. Propidium iodide was excited with a 488 nm laser and emission was detected with a 670 nm longpass (LP) filter. Data were acquired with FACSDiva 6.1.1 (Becton Dickinson) or Prosort 1.5 (Bio-Rad) and analyzed with ModFit LT 3.0 (Verity Software House) or FCS Express 5 (DeNovo) software.

### NHEJ and HR repair reporter assays

The U2OS EJ5 cell line (gift from Dr Jeremy Stark and Prof. Maria Jasin, Memorial Sloan Kettering Cancer Center, NY) was used for assaying NHEJ repair efficiency. Cells were transfected with a plasmid expressing I-SceI or mock transfected with an empty vector for 48 h. TRI-DR-U2OS cells (kind gift from P. Oberdoerffer, Laboratory of Receptor Biology and Gene Expression, NCI/NIH, Bethesda, MD) were used for assaying HR repair efficiency. In this system I-SceI expression was induced by adding 5 μg/ml doxycycline for 48 h. GFP-positive cells were identified and quantified by flow cytometry. The repair efficiency was scored as the percentage of GFP-positive cells. To examine the role of individual genes in DSB repair, cells were treated with siRNAs specifically targeting each gene for 120 h prior to I-SceI expression.

### Statistical analyses

Results are shown as mean±s.e.m. *P*-values were calculated by unpaired, two-tailed Student's *t*-test, or one- or two-way ANOVA with Tukey's corrections for multiple comparisons or Šidák correction. *P<0.05, **P<0.01, ***P<0.001 and ****P<0.0001 for statistical tests, which were performed using GraphPad Prism. *n* indicates the number of independent biological experiments. The number of cells in each experiment is specified in the corresponding figure legend.

## Supplementary Material

Supplementary information

Reviewer comments
