# Supplementary material for: DROSHA is recruited to DNA damage sites by the MRN complex to promote non-homologous end joining
Source: J Cell Sci. 2021 Mar 22;134(6):jcs249706. doi: 10.1242/jcs.249706 (PMC8015226; doi:10.1242/jcs.249706)
Supplement: Supplementary information [file joces-134-249706-s1.pdf]

SUPPLEMENTARY FIGURE 1

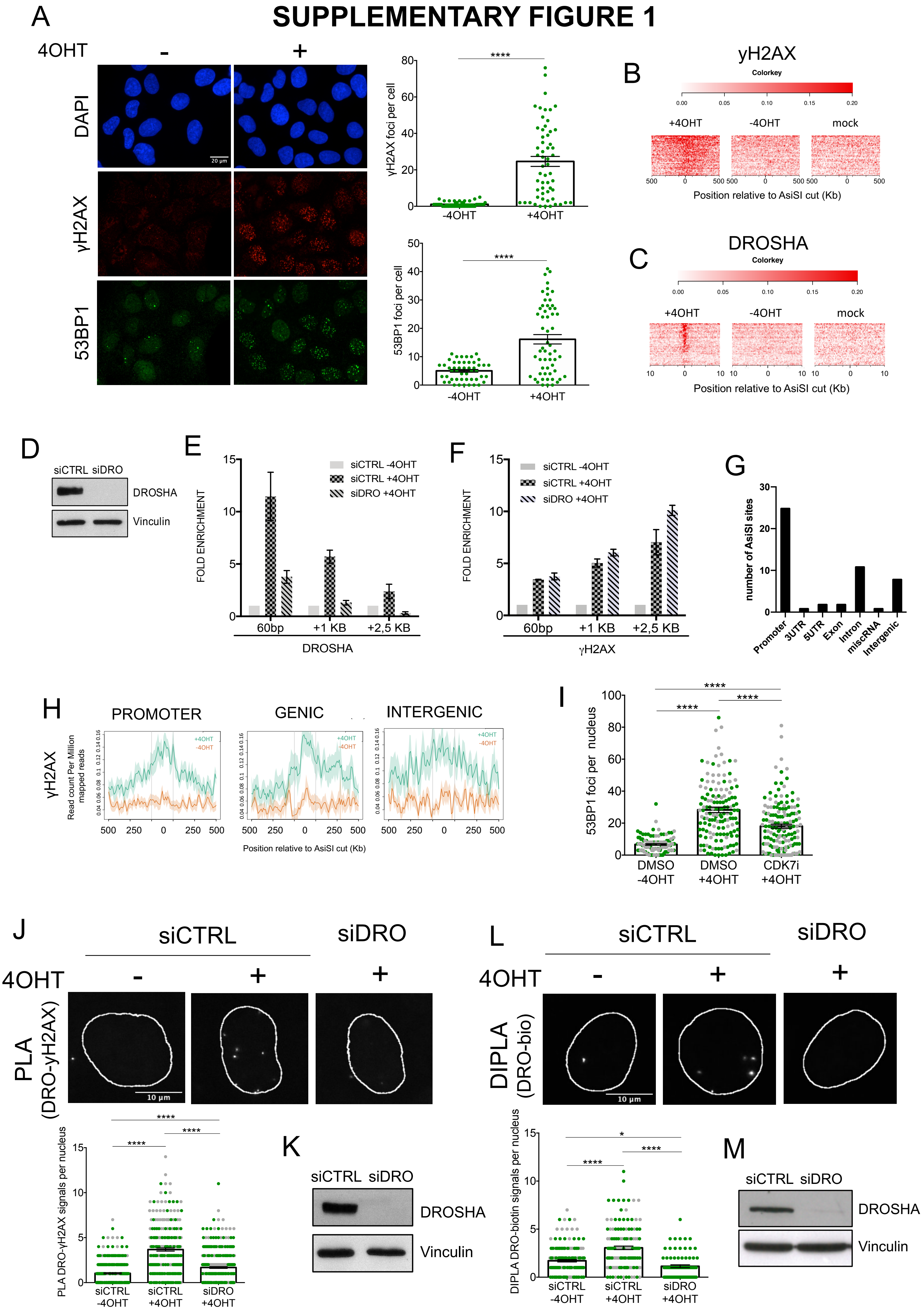

Journal of Cell Science • Supplementary information

Figure S1 | DROSHA accumulates to sites of DNA damage

A) DIvA cells, were co-stained with DAPI, γH2AX and 53BP1, before and after 4OHT treatment (4 h). Scatter plots show the number of γH2AX or 53BP1 foci measured using Cell Profiler software. The black bars represent the mean ± SEM (50 cells, n=1). P-values calculated by Student's t-test. ****P≤0,0001 **B)** Heatmap representation of γH2AX ChIP-seq signal across the 50 AsiSI sites reported in the plot in Figure 1A, sorted by increasing signal, over a 1 Mb window. **C)** Heatmap representation of DROSHA ChIP-seq signal across the 50 AsiSI sites reported in the plot in Figure 1B, sorted by DROSHA increasing signal over a 20 Kb window. **D)** DIvA cells were transfected with non-targeting siRNA (siCTRL) or DROSHA (siDRO). 72h later, KD efficiency was evaluated by western blotting. Vinculin was used as loading control. **E-F)** The bar plot shows the fold enrichment, relative to the uncut (-4OHT), of DROSHA **(E)** and γH2AX **(F)** as detected by ChIP-qPCR in cut (+4OHT), uncut (-4OHT) DIvA cells and cut DIvA cells knock-down for DROSHA (siDRO), with primers matching 60bp, 1Kb or 2,5Kb far from DSB-II (representative experiment, n=2). **G)** The bar plot shows the distribution of the top50 AsiSI sites according to the genetic context in which they have been mapped. **H)** Averaged γH2AX ChIP-seq signal of 8 AsiSI sites positioned in a region annotated as a Promoter, Genic or Intergenic over 1 Mb window and centered at the AsiSI site, are shown for cut (+4OHT, green), ucut (-4OHT, red) or mock (magenta) samples. All the AsiSI sites above are included in the most cut AsiSI sites (Iannelli *et al. 2017)*. **I)** The scatter plot represents the number of 53BP1 foci measured using Cell Profiler automated software in uncut (-4OHT), cut (+4OHT) DIvA cells treated with 1μM CDK7i or mock treated with DMSO. The black bars represent the mean ± SEM (150 cells, n=2). ****P≤0,0001 using one-way ANOVA with multiple comparison. **J)** Representative images of PLA signal of γH2AX-DROSHA proximity in cut (+4OHT) and uncut (-4OHT) DIvA cells and cut DIvA cells knock-down for DROSHA (siDRO). The scatter plot represents the number of PLA signals measured using Cell Profiler automated software. The black bars represent the mean ± SEM (300 cells, n=2). ****P≤0,0001 using by one-way ANOVA with multiple comparison. **K)** DIvA cells were transfected with non-targeting siRNA (siCTRL) or siRNA against DROSHA (siDRO). 72h later, KD efficiency was evaluated by western blotting. Vinculin was used as loading control. **L)** Representative images of DIPLA signal of DNA ends-DROSHA proximity in cut (+4OHT) and uncut (-4OHT) DIvA cells and cut DIvA cells knock-down for DROSHA (siDRO). The scatter plot represents the number of DIPLA signals measured using Cell Profiler automated software. The black bars represent the mean ± SEM (150 cells, n=2). *P≤0,05 ****P ≤0,0001 using one-way ANOVA with multiple comparison. **M)** DIvA cells were transfected with non-targeting siRNA (siCTRL) or siRNA against DROSHA (siDRO). 72h later, KD efficiency was evaluated by western blotting. Vinculin was used as loading control.

Journal of Cell Science • Supplementary information

SUPPLEMENTARY FIGURE 2

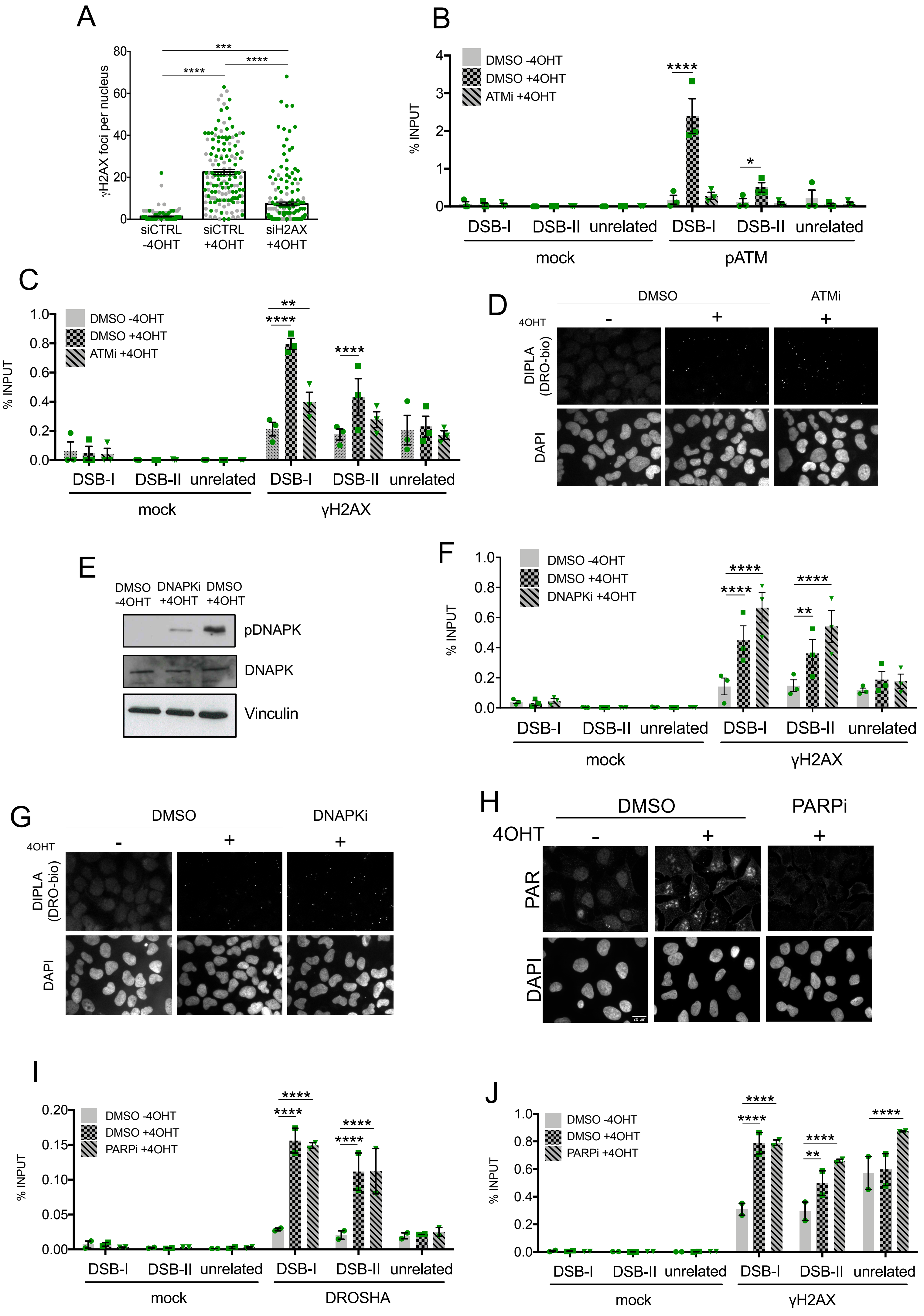

Journal of Cell Science • Supplementary information

Figure S2 | DROSHA recruitment at DNA damage sites occurs independently from H2AX and DDR signalling activation.

A) DIvA cells were transfected with non-targeting siRNA (siCTRL) or siRNA against H2AX (siH2AX). 72h later, KD efficiency was evaluated by immunofluorescence for γH2AX. The scatter plot represents the number of γH2AX foci measured using Cell Profiler automated software. The black bars represent the mean ± SEM (300 cells, n=2). ***P≤0,001 ****P ≤0,0001****P≤0,0001 using one-way ANOVA with multiple comparison. **B-C)** The bar plot shows the percentage of enrichment, relative to the input, of mock (no antibody), pATM **(B)** and γH2AX **(C)** as detected by ChIP-qPCR in cut (+4OHT), uncut (-4OHT) DIvA cells and cut DIvA cells treated with ATM inhibitor (ATMi), with primers matching DSB-I, DSB-II or a genomic region far from any annotated AsiSI sites. Error bars indicate SEM among 3 independent experiments. *P≤0,05 **P≤0,01 ****P ≤0,0001using 2way ANOVA with multiple comparison.

D) Representative images of DIPLA signal of biotin-DROSHA interaction in cut (+4OHT) and uncut (-4OHT) DIvA cells and cut DIvA cells treated with ATM inhibitor (ATMi). E) DIvA cells were mock treated with DMSO or treated with DNAPKi 1h prior to 4OHT induction. DNAPKi efficiency was evaluated by western blotting. Vinculin was used as loading control. F) The bar plot shows the percentage of enrichment, relative to the input, of mock (no antibody) and γH2AX as detected by ChIP-qPCR in cut (+4OHT), uncut (-4OHT) DIvA cells and cut DIvA cells treated with DNAPK inhibitor (DNAPKi), with primers matching DSB-I, DSB-II or a genomic region far from any annotated AsiSI sites. Error bars indicate SEM among 3 independent experiments. **P≤0,01 ****P ≤0,0001 using 2way ANOVA with multiple comparison.

G) Representative images of DIPLA signal of biotin-DROSHA interaction in cut (+4OHT) and uncut (-4OHT) DIvA cells and cut DIvA cells treated with DNAPK inhibitor (DNAPKi).

H) DIvA cells were mock treated with DMSO or treated with PARPi 1h prior to 4OHT induction. PARPi efficiency was evaluated by immunofluorescence for PAR. I-J) The bar plot shows the percentage of enrichment, relative to the input, of mock (no antibody) and DROSHA (I) or γH2AX (J) as detected by ChIP-qPCR in cut (+4OHT), uncut (-4OHT) DIvA cells and cut DIvA cells treated with PARP inhibitor (PARPi), with primers matching DSB-I, DSB-II or a genomic region far from any annotated AsiSI sites. Error bars indicate SEM among 2 independent experiments. **P≤0,01 ****P ≤0,0001 using 2way ANOVA with multiple comparison.

Journal of Cell Science • Supplementary information

SUPPLEMENTARY FIGURE 3

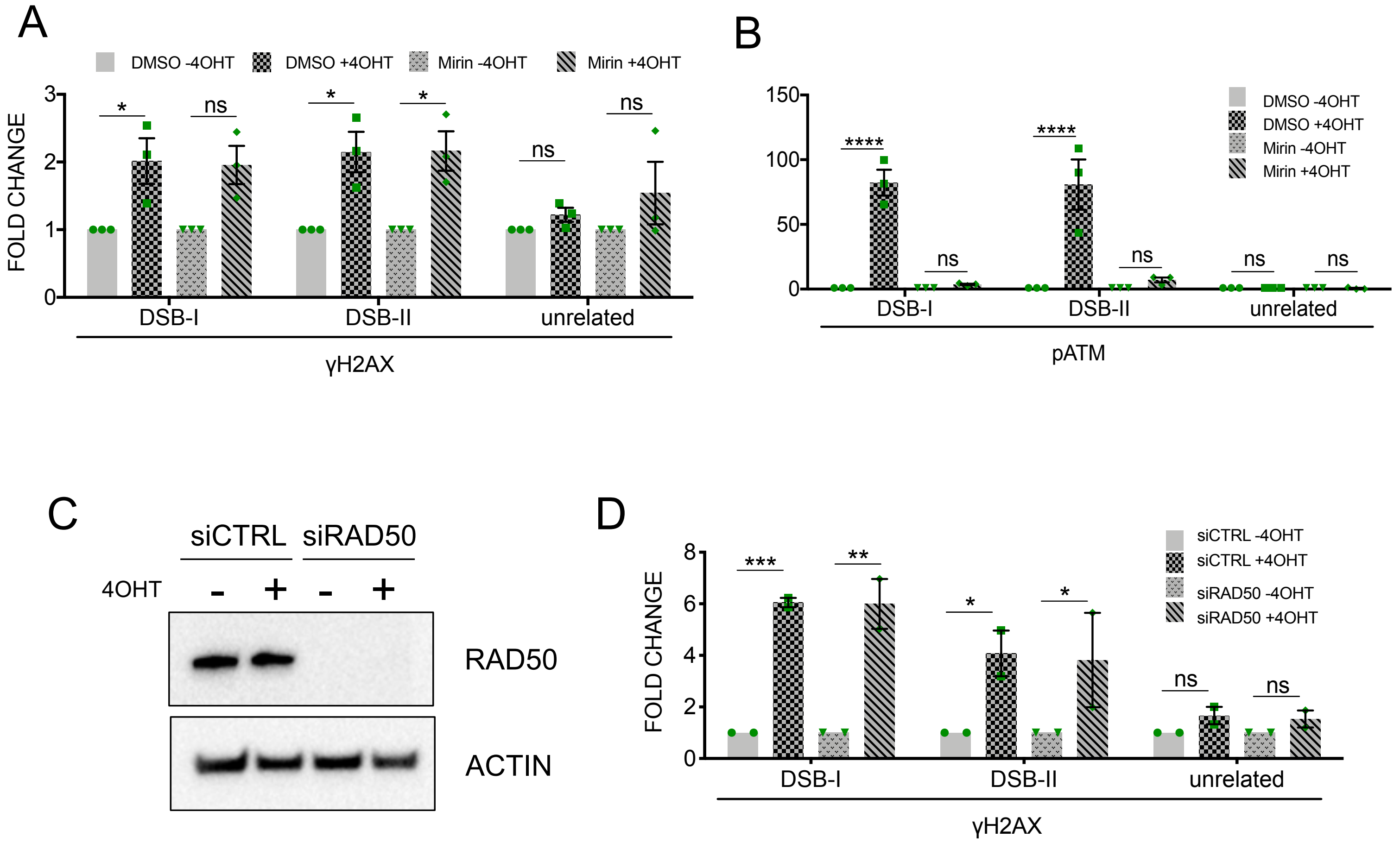

Journal of Cell Science • Supplementary information

Figure S3 | MRN complex support DROSHA recruitment to DNA damage site while ongoing transcription is not required.

A-B) The bar plot shows the fold enrichment, relative to the uncut (-4OHT), of γH2AX **(A)** or pATM **(B)** as detected by ChIP-qPCR in cut (+4OHT) and uncut (-4OHT) DIvA cells treated with Mirin (100 μM) or mock treated with DMSO, with primers matching DSB-I, DSB-II or a genomic region far from any annotated AsiSI sites. Error bars indicate SEM among 3 independent experiments. *P≤0,05 ****P ≤0,0001 using 2way ANOVA with multiple comparison. **C)** DIvA cells were transfected with non-targeting siRNA (siCTRL) or RAD50 (siRAD50). 72h later, KD efficiency was evaluated by western blotting. Vinculin was used as loading control. **D)** The bar plot shows the fold enrichment, relative to the uncut (-4OHT), of γH2AX as detected by ChIP-qPCR in cut (+4OHT) and uncut (-4OHT) DIvA cells knocked down for RAD50 (siRAD50) or mock treated with non-targeting siRNA (siCTRL), with primers matching DSB-I, DSB-II or a genomic region far from any annotated AsiSI sites. Error bars indicate SEM among 2 independent experiments. *P≤0,05 **P≤0,01 ***P≤0,001.

SUPPLEMENTARY FIGURE 4

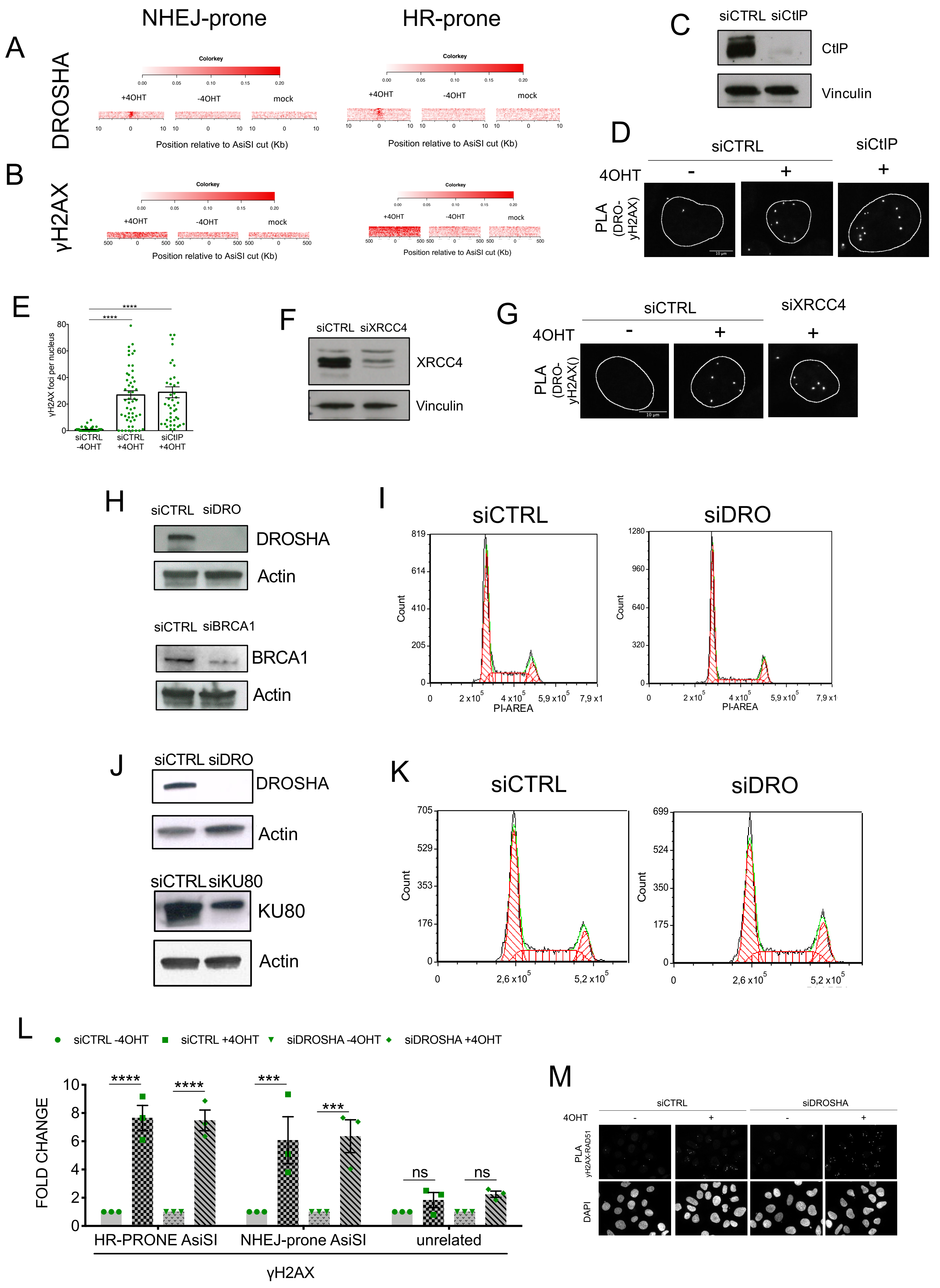

Journal of Cell Science • Supplementary information

Figure S4 | DROSHA recruitment to DSBs occurs throughout the cell cycle and preferentially at NHEJ-prone DSBs.

A-B) Heatmap representation of DROSHA **(A)** or γH2AX **(B)** signal across the AsiSI sites reported in the plot in Figure 4C and 4B, over a 20Kb window or 1 Mb respectively. **C)** DIvA cells were transfected with non-targenting siRNA (siCTRL) or siRNA against CtIP (siCtIP). 72h post transfection, knock-down efficiency was evaluated by western blotting. Vinculin was used as loading control. **D)** Representative images of PLA signal of γH2AX-DROSHA interaction in cut (+4OHT) and uncut (-4OHT) DIvA cells and cut DIvA cells knock down for CtIP (siCtIP). **E)** The scatter plot represents the number of γH2AX foci evaluated by immunofluorescence, measured using Cell Profiler automated software, in DIvA cells transfected with non-targenting siRNA (siCTRL) or siRNA against CtIP (siCtIP). The black bars represent the mean ± SEM (100 cells, n=1). ****P ≤0,0001 using one-way ANOVA with multiple comparison. **F)** DIvA cells were transfected with control siRNA (siCTRL) or siRNA against XRCC4 (siXRCC4). 72h post transfection, knock-down efficiency was evaluated by western blotting. Vinculin was used as loading control. **G)** Representative images of PLA signal of γH2AX-DROSHA interaction in cut (+4OHT) and uncut (-4OHT) DIvA cells and cut DIvA cells knock down for XRCC4 (siXRCC4). **H)** U2OS DR-GFP cells were transfected with non-targenting siRNA (siCTRL), siRNA against DROSHA (siDRO) or siRNA against BRCA1 (siBRCA1). Knock-down efficiency was evaluated by western blotting. Actin was used as loading control. **I)** FACS profiles of U2OS DR-GFP cells transfected with siRNA against DROSHA (siDRO) or mock transfected with non-targeting siRNA (siCTRL). **J)** U2OS EJ5-GFP cells were transfected with non-targeting siRNA (siCTRL), siRNA against DROSHA (siDRO) or siRNA against KU80 (siKU80). Knock-down efficiency was evaluated by western blotting. Actin was used as loading control. **K)** FACS profiles of U2OS EJ5 cells transfected with siRNA against DROSHA or mock transfected with control siRNA (siCTRL). **L)** The bar plot shows the fold enrichment, relative to the uncut (-4OHT), of γH2AX as detected by ChIP-qPCR in cut (+4OHT) and uncut (-4OHT) DIvA cells knocked down for DROSHA (siDROSHA) or mock treated with non-targeting siRNA (siCTRL), with primers matching a NHEJ-prone ASiSI site, a HR-prone AsiSI site or a genomic region far from any annotated AsiSI sites. Error bars indicate SEM among 3 independent experiments. ***P≤0,001 ****P ≤0,0001. **M)** Representative images of PLA signal of γH2AX-RAD51 interaction in cut (+4OHT) and uncut (-4OHT) DIvA cells knock down for DROSHA (siDRO) or mock treated with non-targeting siRNA (siCTRL).

Journal of Cell Science • Supplementary information

Table S1 | List of Antibodies used

Antibody	Company and product ID	Host and Clonality	Applications			
			ChIP/ IP	IF	WB	PLA/ DIPLA
53BP1	Bethyl, A303-906A	goat, polyclonal		1:2000		
53BP1	Novus Biological, 100-305	rabbit, polyclonal	1.5ug			1:2000
γH2AX	Millipore, 05-636	mouse, monoclonal		1:2000		1:2000
γH2AX	Abcam, ab2893	rabbit, polyclonal	2ug			
RNAPII	Abcam, ab817	mouse, monoclonal			1:1000	
Biotin	Sigma-Aldrich, B7653	mouse, monoclonal				1:2000
CtIP	Active Motif, 61142	mouse, monoclonal			1:1000	
DROSHA	Cell Signaling, 3364S	rabbit, monoclonal	5ul		1:1000	1:1000
pATM	Rockland, 200-301-400	mouse, monoclonal	2ug			
RAD50	Millipore 05-525	mouse, monoclonal	5ug		1:1000	
NBS1	Millipore, 04-236	rabbit, polyclonal			1:500	
MRE11	Not commercial	rabbit, polyclonal			1:1000	
pDNAPK	Abcam, ab124918	rabbit, monoclonal			1:2000	
DNAPK	Santa Cruz, Sc5282	mouse, monoclonal			1:1000	
PAR chains	Santa Cruz, Sc56198	mouse, monoclonal		1:100		
Vinculin	Millipore, MAB3574	mouse, monoclonal			1:1000	
Xrcc4	Abcam, ab145	rabbit, polyclonal			1:2000	
BRCA1	Santa Cruz, sc646	rabbit, polyclonal			1:250	
RAD51	Santa Cruz, sc8349	rabbit, polyclonal	2ug			1:500
KU80	Not commercial	Rb			1:10000	
Actin	Sigma-Aldrich, A5441	mouse, monoclonal			1:5000	

Journal of Cell Science • Supplementary information

Table S2 | List of qPCR primers used

NAME	SEQUENCE
DSB I proximal site FW	GATTGGCTATGGGTGTGGAC
DSB I proximal site RV	CATCCTTGCAAACCAGTCCT
DSB II proximal site FW	CCCTGGAGGTAGGTCTGGT
DSB II proximal site RV	CGCACACTCACTGGTTCCT
distal site FW	CCCATCTCAACCTCCACACT
distal site RV	CTTGTCCAGATTCGCTGTGA
DSBII +1Kb FW	AGCCTATGGGATTGGGAAAC
DSBII +1Kb RV	GCCCTTTTCAACCATCTGAC
DSBII +2,5Kb FW	CTCCCCCGAAATCACTTCT
DSBII +2,5Kb RV	AAGAAGGGCAAAGCATGAGA
NHEJ-prone site FW	GGAAGGAGGGGCTACTAGGG
NHEJ-prone site RV	GAAAGCCCCATTCAGTTTGA
HR-prone site FW	CCGCCAGAAAGTTTCCTAGA
HR-prone site FW	CTCACCCTTGCAGCACTTG

Table S3 | List of siRNA used

TARGET	SEQUENCE
**Human DROSHA**	CAACAUAGACUACACGAUU
	CCAACUCCCUCGAGGAUUA
	GGCCAACUGUUAUAGAAUA
	GAGUAGGCUUCGUGACUUA
**Human H2AX**	GGGACGAAGCACUUGGUAA
	CGACUAGAACCUUAGGCAU
	GGAAAGAGCUGAGCCGCUU
	GAACUGGAAUUCUGCAGCU
**CONTROL (non-targeting sequences)**	UGGUUUACAUGUCGACUAA
	UGGUUUACAUGUUGUGUGA
	UGGUUUACAUGUUUUCUGA
	UGGUUUACAUGUUUUCCUA
**Human XRCC4**	UGACCGAGAUCCAGUCUAU
	GAACCCAGUAUAACUCAUU
	CAGCUGAUGUAUACACGUU
	CCUCUUUGAUGAGAUUUAA
**Human CtIP**	GGAGCUACCUCUAGUAUCA
	GAGGUUAUAUUAAGGAAGA
	GAACAGAAUAGGACUGAGU
	GCACGUUGCCCAAAGAUUC
**Human KU80**	GCAUGGAUGUGAUUCAACA
	CGAGUAACCAGCUCAUAAA
	GAGCAGCGCUUUAACAACU
	AAACUUCCGUGUUCUAGUG
**Human BRCA1**	CAACAUGCCCACAGAUCAA
	CCAAAGCGAGCAAGAGAAU
	UGAUAAAGCUCCAGCAGGA
	GAAGGAGCUUUCAUCAUUC
**Human RAD50**	ACAAGGAUCUGGAUAUUUA
	UAACCUCACUGUUGGGGAUA
	GAAUUGAAUCGUAAGCUUA
	GACACAAGGUCAGAAAGUA

Journal of Cell Science • Supplementary information
