# Supplementary material for: DROSHA is recruited to DNA damage sites by the MRN complex to promote non-homologous end joining
Source: J Cell Sci. 2021 Mar 22;134(6):jcs249706. doi: 10.1242/jcs.249706 (PMC8015226; doi:10.1242/jcs.249706)
Supplement: Reviewer comments [file joces-134-249706_review_history.pdf]

Journal of
Cell Science

DROSHA is recruited to DNA damage sites by the MRN complex to promote non-homologous end joining

Matteo Cabrini, Marco Roncador, Alessandro Galbiati, Lina Cipolla, Antonio Maffia, Fabio Iannelli, Simone Sabbioneda, Fabrizio d'Adda di Fagagna and Sofia Francia
DOI: 10.1242/jcs.249706

Editor: Maria Carmo-Fonseca

Review timeline

Original submission:	2 June 2020
Editorial decision:	6 July 2020
First revision received:	4 January 2021
Accepted:	23 January 2021

Original submission

First decision letter

MS ID#: JOCES/2020/249706

MS TITLE: DROSHA is recruited to DNA damage sites by the MRN complex to promote non-homologous end-joining

AUTHORS: Sofia Francia, Fabrizio d'Adda di Fagagna, Matteo Cabrini, Marco Roncador, Alessandro Galbiati, Lina Cipolla, Antonio Maffia, Fabio Iannelli, and Simone Sabbioneda
ARTICLE TYPE: Research Article

We have now reached a decision on the above manuscript.

To see the reviewers' reports and a copy of this decision letter, please go to: https://submit-jcs.biologists.org and click on the 'Manuscripts with Decisions' queue in the Author Area. (Corresponding author only has access to reviews.)

As you will see from their reports, the reviewers' recommendations regarding publication are somewhat mixed. While referee #1 recommends against publication, the other two thought that the work was potentially quite interesting and significant but all also raised a number of concerns that must be dealt with. Please address the issues raised by the three reviewers as thoroughly as possible. If you think that you can deal satisfactorily with the criticisms on revision, I would be pleased to see a revised manuscript. I would then return it to the reviewers.

We are aware that you may currently be unable to access the lab to undertake experimental revisions. If it would be helpful, we encourage you to contact us to discuss your revision in greater detail. Please send us a point-by-point response indicating where you are able to address concerns raised (either experimentally or by changes to the text) and where you will not be able to do so within the normal timeframe of a revision. We will then provide further guidance. Please also note that we are happy to extend revision timeframes as necessary.

Please ensure that you clearly highlight all changes made in the revised manuscript. Please avoid using 'Tracked changes' in Word files as these are lost in PDF conversion.

© 2021. Published by The Company of Biologists under the terms of the Creative Commons Attribution License (https://creativecommons.org/licenses/by/4.0/).

I should be grateful if you would also provide a point-by-point response detailing how you have dealt with the points raised by the reviewers in the 'Response to Reviewers' box. Please attend to all of the reviewers' comments. If you do not agree with any of their criticisms or suggestions please explain clearly why this is so.

Reviewer 1

Advance summary and potential significance to field

In this manuscript Cabrini et al show presence of Drosha at the sites of DNA damage, which is dependent on MRN complex and interaction with Rad50. Lack of Drosha affects NHEJ but stimulates HR.
The requirement of Drosha for DSBs repair, both NHEJ and HR, has been shown previously, as well as its association with DSBs (Lu et al., 2018). Therefore, there is not much novelty in this manuscript. What is molecular mechanism for Drosha's role in DDR is not understood and is not described here.
Hence the manuscript lacks novelty and mechanism and would greatly benefit from further work. In it's current stage it does not provide significant advance to the field of DDR.

Comments for the author

Here are my comments:

In figure 1, as authors suggest that Drosha is localized at promoters, CDS and intergenic regions in similar fashion and therefore it's proximity to chromatin is not dependent on transcription. First of all, it seems that the authors analysed only 8 DSBs? Why not 50? How many fall to promoters, CDS and intergenic regions? Can such small number drive "genome wide" conclusion?
Also some intergenic regions can be transcribed. Therefore, their conclusion is weak. Transcription inhibition of the same region should be used instead.

As mentioned in figure 2 that ATM, DNA-PK and PARP1 are dispensable for recruitment of DROSHA to DSBs. It's better to perform a PLA assay or Immunofluorescence to further verify this phenotype.

Figure 3 has shown that DROSHA could be detected when MRN complex was pulled down. Does DROSHA direct or indirect interact with MRN complex? Have you observed MRN complex when DROSHA pulled down? Besides, it is also possible to knockdown each components of this MRN to further verify the effect of this complex on DROSHA recruitment. Also, it has been mentioned in that 'ongoing transcription is not required for DROSHA recruitment to DNA damage site', when cells were treated CDK7i, to what extent had the transcription been inhibited? A quality control for transcription inhibition efficiency should be performed here.

I have major concern on Figure 4. Firstly, as it stated that 'DROSHA recruitment to DSBs preferentially at NHEJ- prone DSBs', figure 4B showed that DROSHA could be recruited to HR-prone DSBs, what is the function of this protein at those sites upon DNA damage? It will be great if this could be discussed/shown. Also, when HR and NHEJ reporter cassette were applied, DROSHA KD leads to an increase of NHEJ but inhibition of HR, have you observed enrichment of HR factors and less occupancy of NHEJ proteins at those damage sites? Moreover, does it mean that DROSHA could affect the choice of repair pathway? If so, which step is DROSHA involved in and what is the molecular mechanism? Finally, based on the result obtained from HR reporter system, it will be great if you could verify whether the recruitment of downstream HR factors such as RPA and Rad51 will be facilitated upon DROSHA KD, as this should be true if DROSHA KD increases the HR efficiency, this should be added.

Reviewer 2

Advance summary and potential significance to field

In the manuscript entitled "DROSHA is recruited to DNA damage sites by MRN complex to promote non-homologous end joining" the authors demonstrate that DROSHA associated with DSB and its

© 2021. Published by The Company of Biologists under the terms of the Creative Commons Attribution License (https://creativecommons.org/licenses/by/4.0/).

recruitment is one of the upstream events in DDR cascade and independent of ATM and DNA-PKcs kinase activities. The authors show that DROSHA interacts with RAD50 of MRN complex and this interaction is inhibited by a small molecular inhibitor of MRN activity, Mirin. Furthermore, they provide data supporting the fact that DROSHA plays an important role in double strand break repair through NHEJ and not HR. Overall, this is convincing and an interesting study, however it lacks mechanism.

Comments for the author

1. The authors have shown the interaction of RAD50 and DROSHA in Figure 3A, However, their conclusion that DROSHA-RAD50 interaction is reduced upon MIRIN treatment is less convincing.

2. In Figure 3D, the authors have shown the reduction of 53BP1 foci upon Mirin treatment. It will be important to add some high-resolution images to visualize the 53BP1 foci and also, it will be nice to show the ChIP of 53BP1 on the double strand breaks sites with or without Mirin treatment

3. In Figure 4, the authors have nicely demonstrated that DROSHA is important for NHEJ but not for HR.
This conclusion is very convincing, but the mechanism is missing here. Does it interact with some of the factors involved in NHEJ including Ku70/80?

4. The authors should have used two independent siRNAs for DROSHA to rule out the off-target effects for some of the important experiments

Reviewer 3

Advance summary and potential significance to field

In this manuscript, Matteo Cabrini et al. build on previously published findings of the team regarding the role of DROSHA during the DNA damage response to DNA double-strand breaks. Making use of well-established cellular systems and DDR reporters they combine genome-wide tools and targeted approaches to gather a robust collection of data that adequately supports all the authors' conclusions. The main finding of the current study is that DROSHA associates to DSBs in an MRN-dependent manner and this association is necessary for efficient repair of DSBs through NHEJ. The authors show that DROSHA association with broken DNA-ends is independent of transcription levels, ATM, and DNA-PK activity and does not require histone H2AX as a binding platform. Overall the study is very well designed and reports interesting and novel findings that will be of great interest to the scientific community. While no major issues have been identified, a couple of minor comments should be addressed in a revised manuscript.

Comments for the author

Minor Comments:
1. Fig 1D should be complemented with a proper statistical analysis comparing DROSHA levels in the three regions to support the conclusion that DROSHA accumulation is not different. Indeed, they seem quite different (much higher in promoters than in intergenic).

2. The conclusion presented early in the manuscript that DROSHA is recruited to exposed DNA ends regardless of the transcriptional status of the locus where DNA damage is induced finds great support in the data obtained with the CDK7 inhibitor, which are presented later on the manuscript (Fig 3E and 3F). It would help the reader if these data were included in Fig. 1.

3. In Fig 3E and 3F, the label "CDK7" should be replaced by "CDK7i".

4. The manuscript contains several grammar issues that should be corrected.

© 2021. Published by The Company of Biologists under the terms of the Creative Commons Attribution License (https://creativecommons.org/licenses/by/4.0/).

First revision

Author response to reviewers' comments

Reviewer 1 Advance Summary and Potential Significance to Field:
In this manuscript Cabrini et al show presence of Drosha at the sites of DNA damage, which is dependent on MRN complex and interaction with Rad50. Lack of Drosha affects NHEJ but stimulates HR.
The requirement of Drosha for DSBs repair, both NHEJ and HR, has been shown previously, as well as its association with DSBs (Lu et al., 2018). Therefore, there is not much novelty in this manuscript. What is molecular mechanism for Drosha's role in DDR is not understood and is not described here.
Hence the manuscript lacks novelty and mechanism and would greatly benefit from further work. In it's current stage it does not provide significant advance to the field of DDR.

As pointed out by this referee, in the paper (Lu et al., 2018) DROSHA was studied and shown to be recruited at two specific DSBs that falls within promoters of actively transcribed genes. In this study, DROSHA was proposed to be required at site of break for the generation of DNA:RNA hybrid containing pre-existing mRNAs, a structure that, they propose, is functional to both HR and NHEJ DNA repair. In this report however, there is no mechanistic demonstration of what controls DROSHA recruitment to DSB or how DROSHA, or the DNA:RNA hybrid that is generated, can promote both NHEJ and HR which, in fact, are two competing, thus mutually exclusive, DNA repair pathway.

In our manuscript, we adopted a more robust, unbiased, broader, genome-wide approach of DROSHA recruitment as evaluated by ChIP-seq analyses. In this way DROSHA recruitment is studied at DSBs generated within different genomic contexts and upon experimentally-induced transcription inhibition CDK7 inhibitor (THZ1).

In addition, we report on the mechanism of DROSHA recruitment to DNA damage sites. Quite unexpectedly, this does not involve the activation of the upstream DDR kinases or H2AX phosphorylation. Rather, it relies on the primary recruitment of the MRN complex, with which DROSHA can, directly or indirectly, interact – in the revised version of our manuscript, we strengthen this evidence by showing that DROSHA is not recruited to DSB in cells in which MRN has been inactivated by RAD50 knockdown.

Importantly, we also show that, differently from what was reported in (Lu et al., 2018) DROSHA is in fact an inhibitor of HR. This notion is now strengthened in the revised version of this manuscripts by showing that this occurs because DROSHA controls the recruitment at site of break of 53BP1, a key antagonist of DNA end resection (Shibata and Jeggo, 2020) and consequently of HR (Bunting et al., 2010). These novel results are consistent with those published from our group demonstrating by immunofluorescence that DROSHA promotes the secondary recruitment of 53BP1 at DSB (Francia et al., 2016; Francia et al., 2012). In the revised version of the manuscript we delve deep into the consequences of the model porposed and we show, independently by ChIP and PLA approaches, that DROSHA knockdown stimulates the recruitment of the HR factor RAD51 to DSBs. Conversely, we show that treatment with mirin (a MRN inhibitor), or MRN knockdown, by blocking DROSHA recruitment to DSB also impedes 53BP1 accumulation on damaged chromatin, thus strengthening the axis MRN -DROSHA- 53BP1- NHEJ and providing a possible mechanism by which DROSHA contributes to NHEJ and inhibits HR.

In summary, we believe that our revised manuscript is novel because 1) establishes that DROSHA can be recruitment at DSB occurring in different genomic contexts independently from the transcriptional status,as observed by genome wide ChIP-seq unbiased analysis 2) demonstrate that DROSHA rescruitment is controlled by MRN while it is independent on γH2AX as well as the kinase activity of ATM and DNAPK, 3) Proides a novel mechanism of action of DROSHA in DSB repair: by recruiting 53BP1 it fosters NHEJ and inhibitis RAD51 recruitment thus HR.

© 2021. Published by The Company of Biologists under the terms of the Creative Commons Attribution License (https://creativecommons.org/licenses/by/4.0/).

Reviewer 1 Comments for the Author:
Here are my comments:

In figure 1, as authors suggest that Drosha is localized at promoters, CDS and intergenic regions in similar fashion and therefore it's proximity to chromatin is not dependent on transcription. First of all, it seems that the authors analysed only 8 DSBs? Why not 50?

The referee is right: we showed 24 DSB: 8 at gene promoters, 8 at genic and 8 at intergenic genomic positions.

The DIvA cellular system is a powerful tool widley employed to perform genome-wide Chromatin immunoprecipitation (ChIP) followed by next generation sequencing (ChIP-seq) (Aymard et al., 2017; Aymard et al., 2014; Iacovoni et al., 2010; Iannelli et al., 2017) since it allows the concomitant generation of hundreds of DSBs precisely annotated on the human genome .

However, the AsiSI enzyme is sensitive to DNA methylation, reducing the cut efficiency at same loci. To be rigorous in the choice of the AsiSI sites to use for our analysis, we select the 50 AsiSI sites more robustly and reproducibly cut (top 50 AsiSI sites) identified by ChIP-seq analyses for yH2AX. Additionally, we validated this list by comparison with the AsiSI sites obtained by Breaks Labeling In Situ and Sequencing (BLISS) approach (Iannelli et al., 2017; Yan et al., 2017).

Among the top 50 AsiSI sites, 25 hit gene promoters and only 8 fall into intergenic loci (Supplementary Figure 1G, reported below). Being particularly interested in what occurs in the intergenic positions which were not studied in (Lu et al., 2018), we decided to focus on the first 8 DSB also for promoter and genic position. This is why we show comparison of 3 groups of 8 sites and not all 50 ones. In our opinion, choosing to compare an equal number of DSB falling in each genomic context, allowed us to draw correct conclusions on how the chromatin and transcriptional contexcts impacts on DROSHA recruitment. In should be mentioned however that the 25 sites hitting promoter regions are at the top in the ranking for cutting efficiency. This is possibly a fact that contribute to the higher signal observed for DROSHA recruitment in this subgroup.

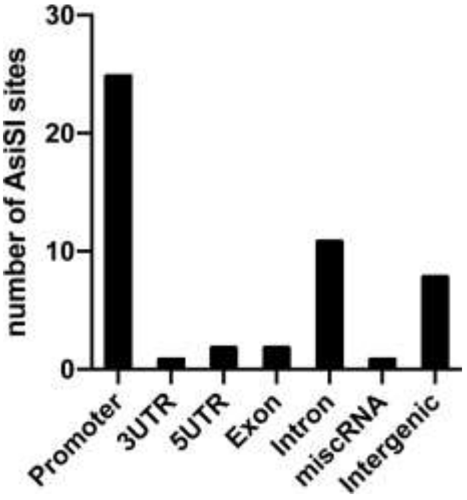

How many fall to promoters, CDS and intergenic regions?

As shown above, Among the Top50 AsiSI sites, 26 are annotated in promoter, 16 in genic positions and only 8 in intergenic regions. This information is now reported Supplementary Figure 1G.

Can such small number drive "genome wide" conclusion?

Our approach was indeed considering the entire genome, however, as already said before, we restrain our comparison analyss to a specific high-confidence subset of AsiSI sites. We believe that this data is still useful to support the notion that DROSHA can be recruited also outside gene's

© 2021. Published by The Company of Biologists under the terms of the Creative Commons Attribution License (https://creativecommons.org/licenses/by/4.0/).

promoters and gene bodies, an information still missing in the present literature.

Neverteless, we understand the criticism of the referee thus we made sure not to use the word "genome- wide" describing this result.

Also some intergenic regions can be transcribed. Therefore, their conclusion is weak.

We totally agree with this referee, intergenic regions can be transcribed at a basal level as a consequence of the pervasive transcription of the geneome (Berretta and Morillon, 2009; Clark et al., 2011; Kapranov et al., 2007). In addition, as we showed already in Supplementary Figure 2L of the paper {Michelini, 2017 #3821}, upon cut also intergenic AsiSI sites induce dilncRNA de-novo transcription activation.

The purpose of our analysis was to show that DROSHA recruitment is not restrained to DSB positioned at promoters of actively transcribed canonical genes. We tryed to better clartified this concept in the text that now has been refrased in:

"This result suggests that promoters tend to recruit more DROSHA than gene body and intergenic site but also indicate that the presence of canonical pre-existing transcription is not a prerequisite for DROSHA association with damaged chromatin."

Transcription inhibition of the same region should be used instead.

Indeed, to demonstrate that transcription (both canonical and de-novo) is not required for DROSHA recruitment at DSBs, we treated DIvA cells with a CDK7 inhibitor (THZ1), thus preventing transcription initiation, shortly prior and during DNA damage induction and we tested by ChIP - qPCR its impact on DROSHA recruitment at DSB. We confirmed that DROSHA recruitment is unaffected at two distinct DSB generated at transcribed regions. We believe that this result supports the notion that ongoing transcription at DSB is not required for DROSHA recruitment. These results are now part of Figure 1G, H and I, as suggested by refree 3.

In addition, to better meet the criticism of this referee, by exploiting a CDK9 (LDC000067) inhibitor, that specifically block transcription elongation, we performed an additional experiment, which we show here for refree only since we consider THZ1 a better tool respect to LDC000067 . In fact THZ1 blocks transcription initiation and not only elongation, and it has been recently shown to have the ability of blocking also dilncRNA transcription (Pessina et al., 2019). However, consistently with the CDK7i result, DROSHA recruitment is unaffected upon treatment with CDK9 inhibitor LCD000067, confirming that ongoing transcription is not a pre-requisite for DROSHA recruitment. The western blot for RNA Pol II and the immunofluorescence for 53BP1 show the efficacy of the treatment, as previously reported in (Michelini et al., 2017).

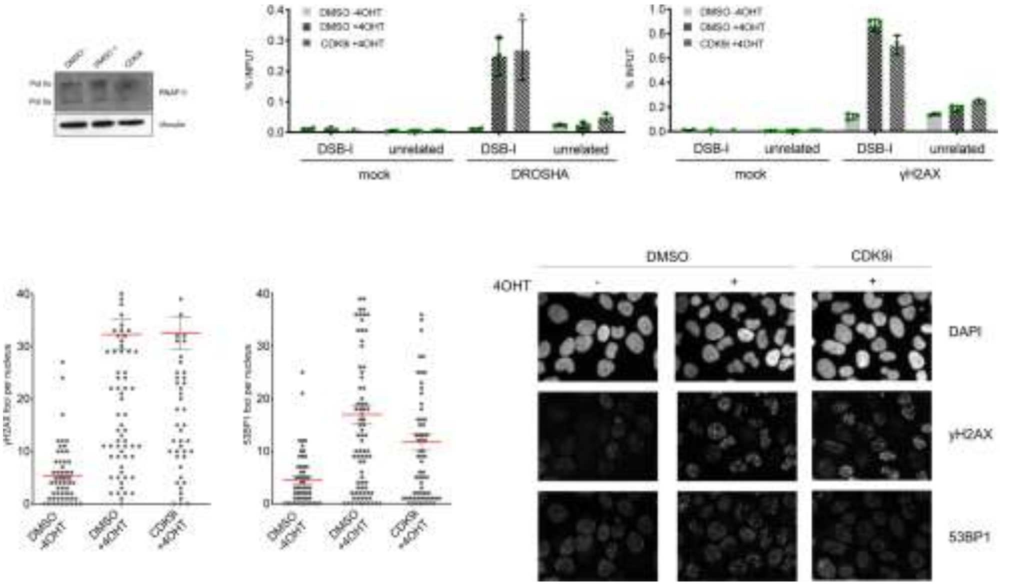

© 2021. Published by The Company of Biologists under the terms of the Creative Commons Attribution License (https://creativecommons.org/licenses/by/4.0/).

As mentioned in figure 2 that ATM, DNA-PK and PARP1 are dispensable for recruitment of DROSHA to DSBs. It's better to perform a PLA assay or Immunofluorescence to further verify this phenotype.

To strengthen our conclusions, we performed additional Di-PLA analyses(Galbiati and d'Adda di Fagagna, 2019), upon treatment with ATM inhibitor and DNAPK inhibitor which are now shown in Figure 2C and E and Supplementary Figure 2D and G. DiPLA is the most accurate method to investigate recruitment of DROSHA at site of break upon inhibition of ATM and DNAPK since these two kinases are responsible for H2AX phosphorylation, thus their inhibition alter the level of ¤H2AX signal. Both immunofluorescence and PLA approaches, in fact, uses yH2AX signal to define DNA damage amount and position. Di-PLA instead allows the detection of the proximity of DROSHA to DNAs exposed upon DSB induction, regardless ¤H2AX signal. As shown in the new figures Figure 2C and E and Supplementary Figure 2D and G, Di-PLA analyses in cells treated with ATM and DNAPK inhibitor, or DMSO as control, support our conclusion that DROSHA is recruited at sites of break independently from ATM and DNAPK kinase activity.

PARylation is not only required for DSB repair but also for single strand break (SSB) repair and base excision repair (BER) (Fleury et al., 2017; Reynolds et al., 2015). Thus treatment with Olaparib induce accumulation of both SSB and DSB. Since we never tested how much PLA and Di-PLA are accurate in distinguishing between SSB and DSB, we believe that ChIP-qPCR at specific DSB induced by AsiSI cut, already present in the manuscript and now shown in Supplemenray Figure 2I, is the most accurate and informative approach to specifically investigate the role played by PARylation in controlling DROSHA recruitment at DSB.

Nevertheless, to further strengthen our conclusions, here we show an immunofluorescence for H2AX and 53BP1 in DIvA cells treated with the PARP inhibitor olaparib. The result clearly shows that treatment with PARP inhibitor doesn't affect the formation of 53BP1 foci, a readout of DROSHA activity in DDR. This result supports the notion that DROSHA recruitment at DSBs is not controlled in a significant manner by PARP1 activity.

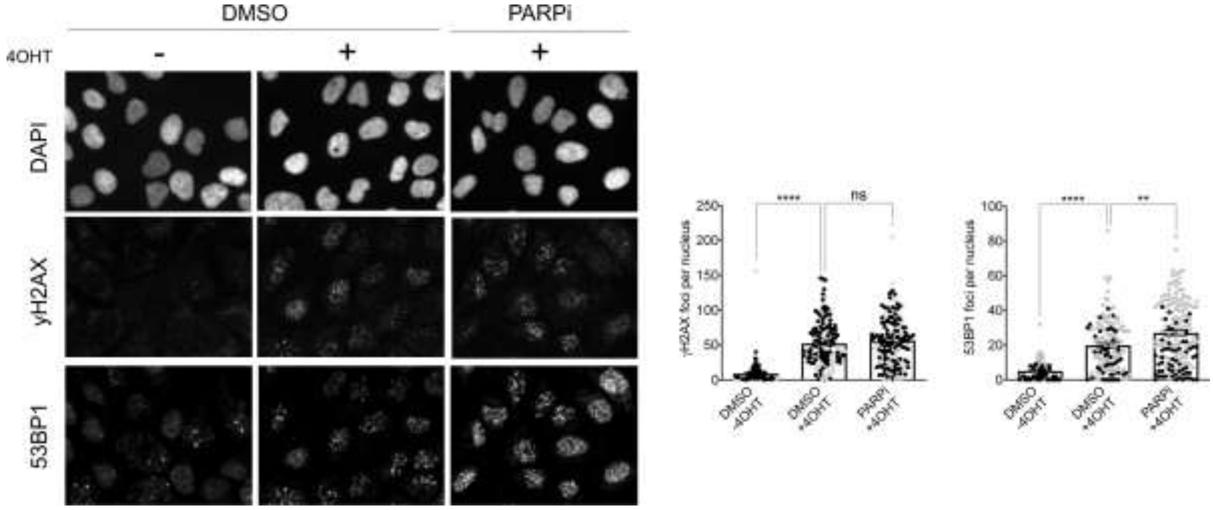

Figure 3 has shown that DROSHA could be detected when MRN complex was pulled down. Does DROSHA direct or indirect interact with MRN complex?

This is a technically difficult experiment to perform since the interaction to test would be between DROSHA, a 150KDa protein, and a trimeric complex that should be reconstituted in vitro by assembly the complex from the three idividual recombinant proteins: RAD50, MRE11 and NBS1. Nevertheless, to answer to this question we obtained recombinant MRN complex assembled in the lab of Petr Cejka, but unfortunately we failed to obtain or generate full length recombinant DROSHA protein.

© 2021. Published by The Company of Biologists under the terms of the Creative Commons Attribution License (https://creativecommons.org/licenses/by/4.0/).

Thus, we used two DROSHA recombinant fragments (one of which was generated and purified from us- fragment 1 and one commercially available-fragment 2) and we tested the interaction with recombinant MRN complex :

1) Fragment 1 spanning amminoacids 1-212 (MW 25KDa) that comprises the Pro-rich and Arg-rich domains of the protein, has been demonstrated to be essential for its interaction with other proteins like CBP80 and the hyperphosphorylated form of RNAPII (Gromak et al., 2013).
2) Fragment 2 spanning amminoacids 1114-1235 (MW 15KDa) that comprirs the RNaseIII domain 2 and mediate the interaction with DGCR8, thus the assembly of Microprocessor (Kwon et al., 2016).

As show in the figure below, unfortunately an interaction between these two DROSHA fragments and the purified recombinant MRN complex was not robustly observed. This may suggest that the interaction is either mediated by other portions of the protein or their post translational modifications (as we observed for the interaction between MDR and RNA PolII in {Michelini, 2017 #3821}) or it is indirect.

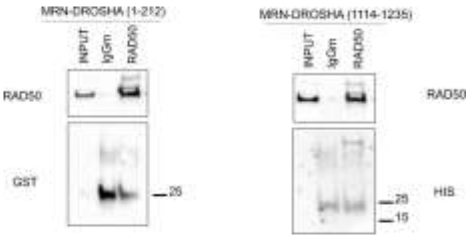

Have you observed MRN complex when DROSHA pulled down?

To test this hypothesis, we initially performed experiments by pulling down endogenous DROSHA despite the fact that DROSHA antibody is not very good for IP. s shown in the following figure we did not see a detectable amount of RAD50 or MRE11 associated with the DROSHA IPed fraction, drifferently from what we observed in RAD50 immunoprecipitation, which reproducibly show a clear interaction with DROSHA (Figure 3A). We also tried to overxpressd a FLAG-tagged version of DROSHA and to IP for Flag. However, also in this setting RAD50 was not detectable in the Flag-DROSHA IPed franction. This may suggest that the fraction of DROSHA interacting with the MRN complex is relatively minor respect to the totality.

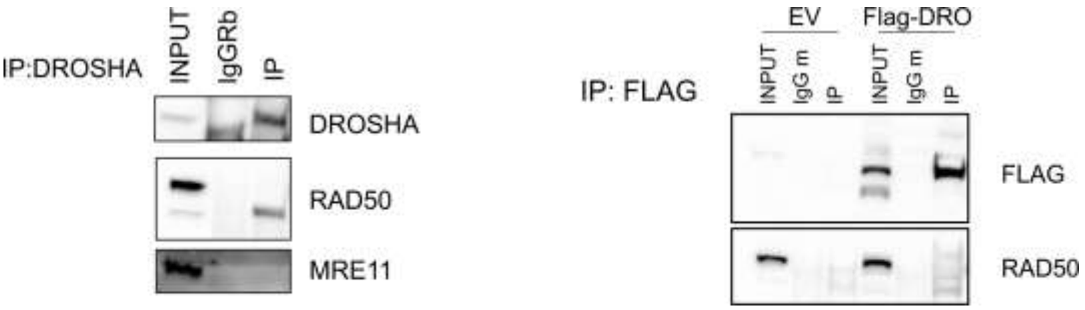

Taking into account these latter negative results, we will agree to reduce the section of our results discussing the MRN-DROSHA interactions, if this is the advice of this Reviewer and our Editor.

Besides, it is also possible to knockdown each components of this MRN to further verify the effect of this complex on DROSHA recruitment.

Indeed, we performed the experiments as suggested by inactivating the MRN complex by RAD50 knockdown and performing ChIP-qPCR for DROSHA in these cells. The complex is unstable in the absence of any of its component (Lamarche et al., 2010). The result obtained is now shown in Figure 3E and F and, as expected, it shows that the inactivation of the MRN complex by depletion

© 2021. Published by The Company of Biologists under the terms of the Creative Commons Attribution License (https://creativecommons.org/licenses/by/4.0/).

of its component RAD50 dramatically reduces DROSHA association with DNA damage site and, as a consequence, also the recruitment of 53BP1 to the same site. This novel set of results further support our conclusion that MRN complex is necessary for DROSHA recruitment to sites of DNA damage.

Also, it has been mentioned in that 'ongoing transcription is not required for DROSHA recruitment to DNA damage site', when cells were treated CDK7i, to what extent had the transcription been inhibited? A quality control for transcription inhibition efficiency should be performed here.

As previously done in literature (Burger et al., 2019), as a quality control for trascriprion inhibition upon CDK7i tratment, we perform a wb for total RNAPII. In Figure 1G of the revised manuscript we now show that RNApolII CTD phosphorylation, corresponding to the active Pol II O status (Gromak et al., 2013) is totally absent in cells treated with CDK7 inhibitor, indicating the efficacy of the treatment.

I have major concern on Figure 4. Firstly, as it stated that 'DROSHA recruitment to DSBs preferentially at NHEJ- prone DSBs', figure 4B showed that DROSHA could be recruited to HR-prone DSBs, what is the function of this protein at those sites upon DNA damage? It will be great if this could be discussed/shown.

Cell populations are asynchronous in these experiments, so all the cells which are in G1 phase of the cell cycle perform NHEJ repair also in HR-prone site. As defined in (Aymard et al., 2014), HR-prone sites are the one that preferentially uses HR repair mechanism when possible (meaning in S-G2 of the cell cycle), nevertheless they can also perform NHEJ in all other conditions. In this study the well known NHEJ factor XRCC4 is also present at HR-prone sites (see figure 1B and C of Aymard et al, 2014). Thus, it is expected that DROSHA, being a NHEJ factor just like XRCC4, is present also at HR-prone sites.

Also, when HR and NHEJ reporter cassette were applied, DROSHA KD leads to an increase of NHEJ but inhibition of HR, have you observed enrichment of HR factors and less occupancy of NHEJ proteins at those damage sites?

In the HR and NHEJ reporter systems, the DSB induction occurs by transient expression of ISceI that targets an engineered artificial locus and cut efficiency is notoriously low. In fact these systems are mostly used in FACS analyses in which the small fraction of GFP positive cells can be counted and compared.

For this reason we decided to test this importat point raised by the referee at endogenous DSB sites in DIvA cells by performing RAD51 ChIP-qPCR upon DROSHA knockdown. In the revised manuscript we now show in Figure 4H and Supplementary figure 4L that DROSHA inactivation indeed promotes the recruitment of RAD51 at HR-prone site, without altering its recruitment elsewhere. In addition, we now we show in Figure 4I and Supplementary Figure 4M that ¤H2AX-RAD51 PLA signals increase upon DROSHA inactivation by siRNA.

Moreover, does it mean that DROSHA could affect the choice of repair pathway? If so, which step is DROSHA involved in and what is the molecular mechanism?

As shown before, we believe that DROSHA influence pathway choice and support repair by NHEJ with the following mechanism: promoting 53BP1 recruitment to damage site, thus inhibiting HR and allow NHEJ to take place.

Finally, based on the result obtained from HR reporter system, it will be great if you could verify whether the recruitment of downstream HR factors such as RPA and Rad51 will be facilitated upon DROSHA KD, as this should be true if DROSHA KD increases the HR efficiency, this should be added.

As described above, we did this experiment in DIvA cells and indeed we can now show that DROSHA knock down stimulate RAD51 recruitment to HR-prone site (Figure 4H and Supplementary figure 4L) . In addition, we showed that ¤H2AX-RAD51 PLA signals increase upon DROSHA inactivation by

© 2021. Published by The Company of Biologists under the terms of the Creative Commons Attribution License (https://creativecommons.org/licenses/by/4.0/).

siRNA (Figure 4I and Supplementary figure 4M) suggesting that DROSHA knockdown indeed facilitates the recruitment of downstream HR factor to break sites.

Reviewer 2 Advance Summary and Potential Significance to Field:
In the manuscript entitled "DROSHA is recruited to DNA damage sites by MRN complex to promote non-homologous end joining" the authors demonstrate that DROSHA associated with DSB and its recruitment is one of the upstream events in DDR cascade and independent of ATM and DNA-PKcs kinase activities. The authors show that DROSHA interacts with RAD50 of MRN complex and this interaction is inhibited by a small molecular inhibitor of MRN activity, Mirin. Furthermore, they provide data supporting the fact that DROSHA plays an important role in double strand break repair through NHEJ and not HR. Overall, this is convincing and an interesting study, however it lacks mechanism.

We thank this referee for defining our study convincing and interesting. The revised manuscript provides more information about the mechanism by which DROSHA controls DNA repair. Indeed we could show that lack of DROSHA recruitment, result in loss of 53BP1 recruitment in cells treated with mirin or knocked down per RAD50 (Figure 3B, D and F). We believe this provide a suggestion of the mechanism by which DROSHA promotes NHEJ while inhibiting HR.

Reviewer 2 Comments for the Author:
1. The authors have shown the interaction of RAD50 and DROSHA in Figure 3A, However, their conclusion that DROSHA-RAD50 interaction is reduced upon MIRIN treatment is less convincing.

To provide more convincing evidence to this referee, we show here additional blots of a biological replicate of the experiment shown in Figure 3A. We hope that this additional independent replicate strengthen the result. Nevertheless we would like to keep the original figure as already shown since in that experiment, all the samples have been loaded adjacently on the same gel.

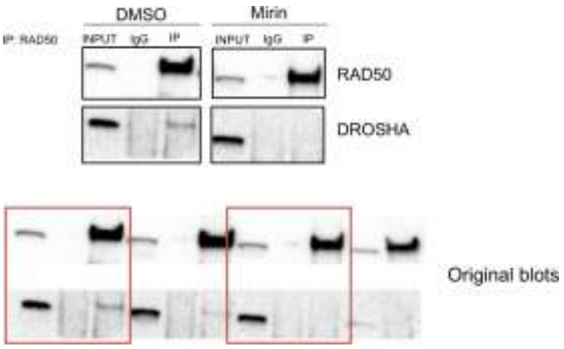

In addition in the revised manuscript we generated more data supporting the role of the MRN complex in promoting DROSHA recruitment to DNA damage site, since we show that not only mirin treatment but also MRN depletion abolishes the recruitment of DROSHA to break sites (Figures 3C, D, E and F). These additional evidences support the notion that MRN is the key factor promoting DROSHA association with break site.

2. In Figure 3D, the authors have shown the reduction of 53BP1 foci upon Mirin treatment. It will be important to add some high-resolution images to visualize the 53BP1 foci and also, it will be nice to show the ChIP of 53BP1 on the double strand breaks sites with or without Mirin treatment

Following the request of this referee, we now included high resolution images of 53BP1 foci in control and mirin treated cells, and the relative quantification by the automated software Cell Profiler in Figure 3B. Moreover, as suggested, we performed additional ChIP analyses for 53BP1 in cells treated with mirin or knocked down for RAD50 and the results obtained fully support the notion that 53BP1 recruitment is affected upon both, mirin treatment and MRN knockdown (now shown in Figure 3C, D, E and F).

3. In Figure 4, the authors have nicely demonstrated that DROSHA is important for NHEJ but

© 2021. Published by The Company of Biologists under the terms of the Creative Commons Attribution License (https://creativecommons.org/licenses/by/4.0/).

not for HR. This conclusion is very convincing, but the mechanism is missing here. Does it interact with some of the factors involved in NHEJ including Ku70/80?

We tested this hypothesis by probing for DROSHA presence the IPed fraction of KU80 in 293T cells. As shown here below, we could not detect any DROSHA associate with KU80.

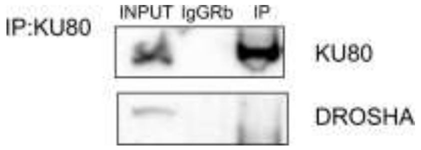

We believe that the importance of DROSHA in NHEJ, and not in HR, strictly depends in its role in supporting 53BP1 recruitment to break site, thus inhibiting DNA end resection and HR.

4. The authors should have used two independent siRNAs for DROSHA to rule out the off-target effects for some of the important experiments

For these experiments we used a pool of 4 siRNA from Dharmacon that have been optimized to avoid off target effects. Here below we show what is the description of this patented technology. Therefore we believe that we are already using the best tool to perform DROSHA knockdown experiments.

[an image provided to the reviewers has been removed. The information shown in the image can be found at https://horizondiscovery.com/en/gene-modulation/knockdown/sirna/products/on-targetplus-sirna-reagents]

In addition we show here that 53BP1 foci are lost also upon DROSHA knockdown by using two other silencing approaches with different targeting sequences: viral infection of an shRNA vector against DROSHA and siRNA pool of old generation-siGENOME from Dharmacon. We believe these result disprove the hypothesis that the result obtained are in fact due to off-target.

© 2021. Published by The Company of Biologists under the terms of the Creative Commons Attribution License (https://creativecommons.org/licenses/by/4.0/).

53BP1 foci are reduced in DIvA cells infected with lentiviral vector expressing shRNA against DROSHA

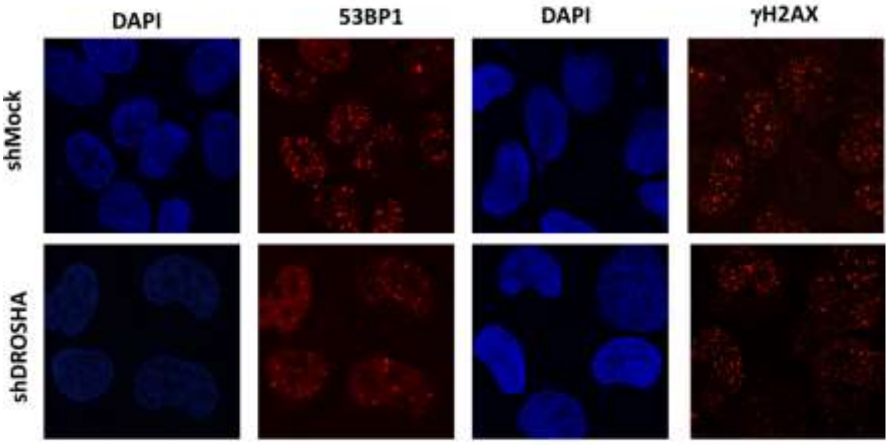

53BP1 foci in induced DIvA cells knocked-down for DROSHA with Dharmacon si GENOME siRNAs

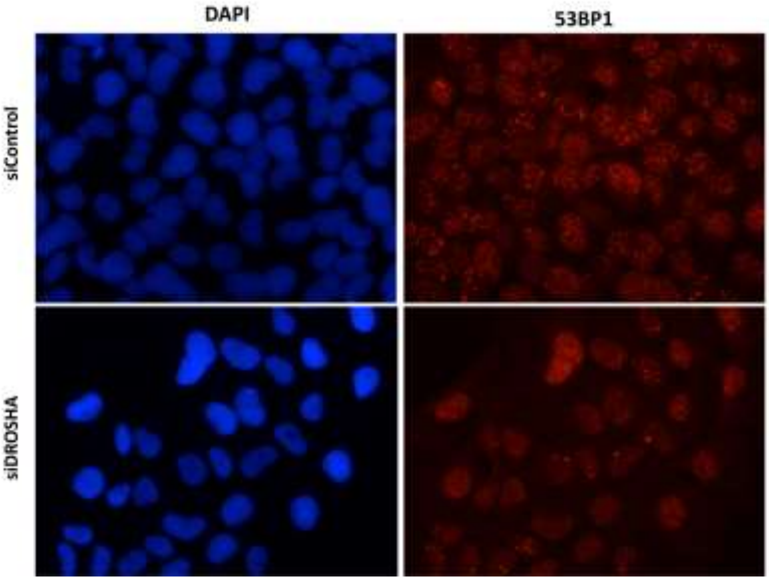

Reviewer 3 Advance Summary and Potential Significance to Field:
In this manuscript, Matteo Cabrini et al. build on previously published findings of the team regarding the role of DROSHA during the DNA damage response to DNA double-strand breaks. Making use of well-established cellular systems and DDR reporters they combine genome-wide tools and targeted approaches to gather a robust collection of data that adequately supports all the authors' conclusions. The main finding of the current study is that DROSHA associates to DSBs in an MRN-dependent manner and this association is necessary for efficient repair of DSBs through NHEJ. The authors show that DROSHA association with broken DNA-ends is independent of transcription levels, ATM, and DNA-PK activity and does not require histone H2AX as a binding platform. Overall the study is very well designed and reports interesting and novel findings that will be of great interest to the scientific community. While no major issues have been identified, a couple of minor comments should be addressed in a revised manuscript.

Reviewer 3 Comments for the Author:
Minor Comments:
1. Fig 1D should be complemented with a proper statistical analysis comparing DROSHA levels in the three regions to support the conclusion that DROSHA accumulation is not

© 2021. Published by The Company of Biologists under the terms of the Creative Commons Attribution License (https://creativecommons.org/licenses/by/4.0/).

different. Indeed, they seem quite different (much higher in promoters than in intergenic).

All our ChIP-seq analyses uses Chi-Square as statistical tool for peak calling of induced samples versus uninduced one. This was done also in the case of sub groups of cut sites falling into promoters, genic and intergenic loci. So, widely statistical tolls have been applied for our comparison fChIP-seq picks around the genome.

But we indeed agree with this referee that there is a difference between the enrichment of DROSHA observed in promoters respect to genic and intergenic AsiSI sites. Possibly we were not clear enought in the text of the manuscript thus we changed the wording in the following way: "This result suggests that promoters tend to recruit more DROSHA than gene body and intergenic site but also indicate that the presence of canonical pre-existing transcription is not a prerequisite for DROSHA association with damaged chromatin."

What we want to highlight here is that there is a statistical significant recruitment of DROSHA at cut sites that can occur also at untrascribed regions.

2. The conclusion presented early in the manuscript that DROSHA is recruited to exposed DNA ends regardless of the transcriptional status of the locus where DNA damage is induced finds great support in the data obtained with the CDK7 inhibitor, which are presented later on the manuscript (Fig 3E and 3F). It would help the reader if these data were included in Fig. 1.

Indeed we agree with this referee and we did that. We positioned the results obtained with CDK7 inhibitor in Figure 1 as suggested.. For referees only we also show that treatment with CDK9 inhibitor, another transcriptional inhibitor, give the same result in term of unaffected DROSHA recruitment to break site hitting transcribed loci (Se reply to referee 1 above).

3. In Fig 3E and 3F, the label "CDK7" should be replaced by "CDK7i".

Thank you, we corrected that.

4. The manuscript contains several grammar issues that should be corrected.

To solve this problem, we asked a native speaker to read the revised manuscript.

References

Aymard, F., Aguirrebengoa, M., Guillou, E., Javierre, B.M., Bugler, B., Arnould, C., Rocher, V., Iacovoni, J.S., Biernacka, A., Skrzypczak, M., *et al.* (2017). Genome-wide mapping of long-range contacts unveils clustering of DNA double-strand breaks at damaged active genes. Nat Struct Mol Biol *24*, 353-361.

Aymard, F., Bugler, B., Schmidt, C.K., Guillou, E., Caron, P., Briois, S., Iacovoni, J.S., Daburon, V., Miller, K.M., Jackson, S.P., *et al.* (2014). Transcriptionally active chromatin recruits homologous recombination at DNA double-strand breaks. Nature structural & molecular biology *21*, 366- 374.

Berretta, J., and Morillon, A. (2009). Pervasive transcription constitutes a new level of eukaryotic genome regulation. EMBO reports *10*, 973-982.

Bunting, S.F., Callen, E., Wong, N., Chen, H.T., Polato, F., Gunn, A., Bothmer, A., Feldhahn, N., Fernandez-Capetillo, O., Cao, L., *et al.* (2010). 53BP1 inhibits homologous recombination in Brca1-deficient cells by blocking resection of DNA breaks. Cell *141*, 243-254.

Burger, K., Schlackow, M., and Gullerova, M. (2019). Tyrosine kinase c-Abl couples RNA polymerase II transcription to DNA double-strand breaks. Nucleic Acids Res *47*, 3467-3484.

Clark, M.B., Amaral, P.P., Schlesinger, F.J., Dinger, M.E., Taft, R.J., Rinn, J.L., Ponting, C.P., Stadler, P.F., Morris, K.V., Morillon, A., *et al.* (2011). The reality of pervasive transcription. PLoS

© 2021. Published by The Company of Biologists under the terms of the Creative Commons Attribution License (https://creativecommons.org/licenses/by/4.0/).

biology *9*, e1000625; discussion e1001102.

Fleury, H., Carmona, E., Morin, V.G., Meunier, L., Masson, J.Y., Tonin, P.N., Provencher, D., and Mes- Masson, A.M. (2017). Cumulative defects in DNA repair pathways drive the PARP inhibitor response in high-grade serous epithelial ovarian cancer cell lines. Oncotarget *8*, 40152-40168.

Francia, S., Cabrini, M., Matti, V., Oldani, A., and d'Adda di Fagagna, F. (2016). DICER, DROSHA and DNA damage response RNAs are necessary for the secondary recruitment of DNA damage response factors. J Cell Sci *129*, 1468-1476.

Francia, S., Michelini, F., Saxena, A., Tang, D., de Hoon, M., Anelli, V., Mione, M., Carninci, P., and d'Adda di Fagagna, F. (2012). Site-specific DICER and DROSHA RNA products control the DNA-damage response. Nature *488*, 231-235.

Galbiati, A., and d'Adda di Fagagna, F. (2019). DNA Damage In Situ Ligation Followed by Proximity Ligation Assay (DI-PLA). Methods Mol Biol *1896*, 11-20.

Gromak, N., Dienstbier, M., Macias, S., Plass, M., Eyras, E., Caceres, J.F., and Proudfoot, N.J. (2013). Drosha regulates gene expression independently of RNA cleavage function. Cell Rep *5*, 1499-1510.

Iacovoni, J.S., Caron, P., Lassadi, I., Nicolas, E., Massip, L., Trouche, D., and Legube, G. (2010). High- resolution profiling of gammaH2AX around DNA double strand breaks in the mammalian genome. The EMBO journal *29*, 1446-1457.

Iannelli, F., Galbiati, A., Capozzo, I., Nguyen, Q., Magnuson, B., Michelini, F., D'Alessandro, G., Cabrini, M., Roncador, M., Francia, S., *et al.* (2017). A damaged genome's transcriptional landscape through multilayered expression profiling around in situ-mapped DNA double-strand breaks. Nat Commun *8*, 15656.

Kapranov, P., Cheng, J., Dike, S., Nix, D.A., Duttagupta, R., Willingham, A.T., Stadler, P.F., Hertel, J., Hackermuller, J., Hofacker, I.L., *et al.* (2007). RNA maps reveal new RNA classes and a possible function for pervasive transcription. Science *316*, 1484-1488.

Kwon, S.C., Nguyen, T.A., Choi, Y.G., Jo, M.H., Hohng, S., Kim, V.N., and Woo, J.S. (2016). Structure of Human DROSHA. Cell *164*, 81-90.

Lamarche, B.J., Orazio, N.I., and Weitzman, M.D. (2010). The MRN complex in double-strand break repair and telomere maintenance. FEBS Lett *584*, 3682-3695.

Lu, W.T., Hawley, B.R., Skalka, G.L., Baldock, R.A., Smith, E.M., Bader, A.S., Malewicz, M., Watts, F.Z., Wilczynska, A., and Bushell, M. (2018). Drosha drives the formation of DNA:RNA hybrids around DNA break sites to facilitate DNA repair. Nat Commun *9*, 532.

Michelini, F., Pitchiaya, S., Vitelli, V., Sharma, S., Gioia, U., Pessina, F., Cabrini, M., Wang, Y., Capozzo, I., Iannelli, F., *et al.* (2017). Damage-induced lncRNAs control the DNA damage response through interaction with DDRNAs at individual double-strand breaks. Nat Cell Biol *19*, 1400-1411.

Pessina, F., Giavazzi, F., Yin, Y., Gioia, U., Vitelli, V., Galbiati, A., Barozzi, S., Garre, M., Oldani, A., Flaus, A., *et al.* (2019). Functional transcription promoters at DNA double-strand breaks mediate RNA- driven phase separation of damage-response factors. Nat Cell Biol *21*, 1286-1299.

Reynolds, P., Cooper, S., Lomax, M., and O'Neill, P. (2015). Disruption of PARP1 function inhibits base excision repair of a sub-set of DNA lesions. Nucleic Acids Res *43*, 4028-4038.

Shibata, A., and Jeggo, P.A. (2020). Roles for the DNA-PK complex and 53BP1 in protecting ends from resection during DNA double-strand break repair. J Radiat Res *61*, 718-726.

© 2021. Published by The Company of Biologists under the terms of the Creative Commons Attribution License (https://creativecommons.org/licenses/by/4.0/).

<u>Second decision letter</u>

MS ID#: JOCES/2020/249706

MS TITLE: DROSHA is recruited to DNA damage sites by the MRN complex to promote non-homologous end-joining

AUTHORS: Sofia Francia, Fabrizio d'Adda di Fagagna, Matteo Cabrini, Marco Roncador, Alessandro Galbiati, Lina Cipolla, Antonio Maffia, Fabio Iannelli, and Simone Sabbioneda
ARTICLE TYPE: Research Article

I am happy to tell you that your manuscript has been accepted for publication in Journal of Cell Science, pending standard ethics checks.

<u>Reviewer 1</u>

Advance summary and potential significance to field

Although still missing detailed mechanism, this revised manuscript is improved and provides contribution to the field of DDR. The role of Drosha at the breaks needs to be explored further in future studies and the data shown here could form a stepping stone for such research. Therefore I recommend this manuscript for publication.

Comments for the author

The authors addressed all my comments and their additional experiments and modifications make the manuscript acceptable for publication.

<u>Reviewer 2</u>

Advance summary and potential significance to field

In the manuscript entitled “DROSHA is recruited to DNA damage sites by MRN complex to promote non-homologous end joining” the authors demonstrate that DROSHA associated with DSB and its recruitment is one of the upstream events in DDR cascade and independent of ATM and DNA-PKcs kinase activities. The authors show that DROSHA interacts with RAD50 of MRN complex and this interaction is inhibited by a small molecular inhibitor of MRN activity, Mirin.
Furthermore, they provide data supporting the fact that DROSHA plays an important role in double strand break repair through NHEJ and not HR.

Comments for the author

The authors have addressed all the questions satisfactorily in this revised version of manuscript entilted "DROSHA is recruited to DNA damage sites by the MRN complex to promote non-homologus end joining".

© 2021. Published by The Company of Biologists under the terms of the Creative Commons Attribution License (https://creativecommons.org/licenses/by/4.0/).
